# DJ-1 (Park7) affects the gut microbiome, metabolites and the development of innate lymphoid cells (ILCs)

**DOI:** 10.1038/s41598-020-72903-w

**Published:** 2020-09-30

**Authors:** Yogesh Singh, Christoph Trautwein, Achal Dhariwal, Madhuri S. Salker, Md Alauddin, Laimdota Zizmare, Lisann Pelzl, Martina Feger, Jakob Admard, Nicolas Casadei, Michael Föller, Vivek Pachauri, David S. Park, Tak W. Mak, Julia-Stefanie Frick, Diethelm Wallwiener, Sara Y. Brucker, Florian Lang, Olaf Riess

**Affiliations:** 1 Institute of Medical Genetics and Applied Genomics, Tübingen University, Calwerstraße 7, 72076 Tübingen, Germany; 2 Department of Preclinical Imaging and Radiopharmacy, Werner Siemens Imaging Center (WSIC), Tübingen University, Röntgenweg 13, 72076 Tübingen, Germany; 3 Department of Oral Biology, University of Oslo, Oslo, Norway; 4 Research Institute of Women’s Health, Tübingen University, Calwerstraße 7/6, 72076 Tübingen, Germany; 5 Department of Vegetative Physiology, Tübingen University, Wilhelmstraße 56, 72076 Tübingen, Germany; 6 Clinical Transfusion Medicine Centre, Tübingen University, Otfried-Müller-Straße 4/1, 72076 Tübingen, Germany; 7 Department of Physiology, University of Hohenheim, Garbenstraße 30, 70599 Stuttgart, Germany; 8 Institute of Materials in Electrical Engineering 1, RWTH Aachen University, Aachen, Germany; 9 Health Research Innovation Centre, Hotchkiss Brain Institute, 3330 Hospital Drive NW, Calgary, Alberta T2N 4N1 Canada; 10 Campbell Family Institute for Breast Cancer Research, Ontario Cancer Institute, UHN, 620 University Ave, Toronto, M5G 2C1 Canada; 11 Institute for Medical Microbiology and Hygiene, Tübingen University, Elfriede-Aulhorn-Straße 6, 72076 Tübingen, Germany

**Keywords:** Innate lymphoid cells, Microbiome

## Abstract

The proper communication between gut and brain is pivotal for the maintenance of health and, dysregulation of the gut-brain axis can lead to several clinical disorders. In Parkinson’s disease (PD) 85% of all patients experienced constipation many years before showing any signs of motor phenotypes. For differential diagnosis and preventive treatment, there is an urgent need for the identification of biomarkers indicating early disease stages long before the disease phenotype manifests. DJ-1 is a chaperone protein involved in the protection against PD and genetic mutations in this protein have been shown to cause familial PD. However, how the deficiency of DJ-1 influences the risk of PD remains incompletely understood. In the present study, we provide evidence that DJ-1 is implicated in shaping the gut microbiome including; their metabolite production, inflammation and innate immune cells (ILCs) development. We revealed that deficiency of DJ-1 leads to a significant increase in two specific genera/species, namely *Alistipes* and *Rikenella*. In DJ-1 knock-out (DJ-1^-/-^) mice the production of fecal calprotectin and MCP-1 inflammatory proteins were elevated. Fecal and serum metabolic profile showed that malonate which influences the immune system was significantly more abundant in DJ-1^−/−^ mice. DJ-1 appeared also to be involved in ILCs development. Further, inflammatory genes related to PD were augmented in the midbrain of DJ-1^−/−^ mice. Our data suggest that metabolites and inflammation produced in the gut could be used as biomarkers for PD detection. Perhaps, these metabolites and inflammatory mediators could be involved in triggering inflammation resulting in PD pathology.

## Introduction

Parkinson’s disease (PD) is the most common movement disorder and the second most prevalent neurodegenerative disease in humans^1^. Clinically, PD patients suffer from resting tremor, rigidity, bradykinesia and altered gait^1^. PD is an incurable neurodegenerative disease distinguished by the loss of neurons predominantly in the *substantia nigra pars compacta (SNpc)* region in the mid brain and the presence of Lewy bodies in the surviving neurons. However, the exact cause of PD and how the disease process is triggered remains incompletely understood^2^. Most of the PD cases are sporadic, however, several rare genetic forms of the disease have been identified that have contributed prominently to our understanding of the mechanisms underlying disease pathogenesis^3^. Within Lewy bodies, the presence of intracellular protein inclusions are mainly comprised of misfolded alpha-Synuclein (α-Syn), which has also been shown to be genetically linked to familial and sporadic forms of PD^4,5^.

In addition to α-Syn-related genetic links with PD, several other mutations such as PARK7 (DJ-1), Parkin, UCH-L1, Pink1 and dardarin genes account for sporadic cases with early-onset recessive PD^6,7^. DJ-1 is a small ubiquitously expressed protein that is implicated in several pathways associated with PD pathogenesis^3^. The DJ-1 protein is encoded by the PARK7 gene and comprises of 189 amino acids^8^. DJ-1 is localized primarily in the cytoplasm, however it can also be found in the nucleus and is linked with mitochondria^8^. DJ-1 is involved in several cellular functions, serving as an oxidative stress sensor (via a cysteine residue at position 106, C106), a protein chaperone, a protease, an RNA-binding protein, a transcription regulator, a regulator of mitochondria function and a regulator of autophagy^9^. It is still not clear which of these processes are responsible for DJ-1-dependent pathogenesis in PD.

Previous studies revealed that DJ-1 is involved in the modulation, aggregation, and toxicity of α-Syn^8,10–12^. Double transgenic mice for DJ-1 deficiency and α-Syn expression (expressing pathogenic Ala53Thr human a-Syn) called M83-DJ-1 null mice, revealed that onset of disease and pathological changes were not different when compared with single transgenic M83 mice line and concluded that α-Syn and DJ-1 mutation may lead to PD via independent mechanisms^13^. However, a recent in vitro study revealed that DJ-1 directly binds monomeric/oligomeric α-Syn and showed that DJ-1 interacts with α-Syn in living cells^8^. Nevertheless, according to functional genomics approaches in both Drosophila and Yeast models suggest that overexpression of DJ-1 has protective mechanisms against neuronal cell dysfunctions and death^8^. Dopaminergic (DA) neurons numbers in the *SNpc* region and fibre densities and dopamine levels in the striatum were reported to be normal in DJ-1 knock-out (DJ-1^−/−^) mice. Furthermore, enhanced striatal denervation and DA neuron loss was induced by 1-methy-4-phenyl-1,2,3,6-tetrahydropyridine (MPTP) together with amphetamine in DJ-1^−/−^ mice^7^. Additionally, DJ-1^−/−^ mice, which were backcrossed with C57BL/6 mice (known as DJ-1C57^−/−^) suffered from early-onset unilateral loss of DA neurons in their *SNpc*, progressing to bilateral degeneration of the nigrostriatal axis with aging as well as mild motor behaviour deficits at aged time points^14^. The authors suggested that the DJ1-C57 model effectively recapitulates the early stages of PD and allow study of the preclinical aspects of neurodegeneration^14^. Our own studies with DJ-1^−/−^ mice also suggested that DJ-1 protein is involved in the regulation of adaptive immune CD4^+^ T cells and their development and functions by modifying of sodium hydrogen exchanger 1 (NHE1) and ROS formation^15,16^. One should keep in mind that different DJ-1^−/−^ mice lines with different background could be more or less prone to develop neurodegeneration and in additive to genetic changes additional environmental and unknown factors may play a crucial role for the disease development in DJ-1^−/−^ mice. One of the factors could be the gut microbiome which could cause the resistant phenotype. Thus, studying the role of the gut microbiome in the context of disease development in the DJ-1^−/−^ mice model is warranted to understand the PD pathophysiology and to develop novel tools for pre-clinical studies.

Trillions of bacteria reside in the gut named jointly the gut microbiome. They are important for normal functioning of the intestine^17^. Recent advances in metagenomics uncover the profound impact of the microbiota on neurodevelopment and diseases of the central nervous system (CNS)^18,19^. An essential function of the gastrointestinal tract (GIT) is to perceive and react to external signals such as environment, food and xenobiotics^20^. Studies from germ-free (GF) and antibiotic-treated mice suggested that bacteria are pivotal in hippocampal neurogenesis as well as spatial and object recognition^21^. In mice, antibiotics treatment changed transiently the microbiota, and as a result increased expression of the brain-derived neurotropic factor in the hippocampus and enhanced exploratory behavior^22^. Further studies suggested that the microbiota promotes enteric and circulating serotonin (5-hydroxytryptamine, 5-HT) production from colonic enterochromaffin cells, modulates the GIT mobility, platelet functions^23^, affects anxiety, hyperactivity and cognition^24,25^. Dysbiosis (alterations to the microbial composition) of the microbiome has not only been described in murine, but also in humans with neurological diseases^26^. For example, fecal and mucosa-associated gut microbes are different between PD patients and healthy controls^27–31^. The Thy1-α-Syn [ASO] transgenic PD mouse model study also suggested that PD patient-derived microbiota have adverse effect on the Parkinson’s pathogenesis in this mouse model including the accumulation of α-Syn and change in the motor phenotype^32^. Several other mouse studies also suggested the gut dysbiosis in chemically induced toxins and other PD models^32–38^. Human studies from fecal metabolites suggested that PD patients have reduced short chain fatty acids (SCFAs), which are the metabolic products of certain gut bacteria^31^. However, how bacterial produced gut metabolites are affecting the neurodegenerative diseases are not understood.

Immune cells are capable of engaging in direct communication with enteric neurons^18,20^. The extent of the functional impact of neuro-immune synapses is not yet clear. However, published studies advocated that activated immune cells can temper neuronal activity via the release of neurotransmitters, metabolites and cytokines^19,39,40^. Based on the common occurrence of GIT symptoms in PD, dysbiosis among PD patients, and evidence that the microbiota impacts on CNS function, we hypothesized that the DJ-1 protein could also be involved in the regulation of the gut microbiome and inflammation. Herein, we report that the microbiota composition is dysregulated, innate immunity altered, inflammation increased (colon, feces, and brain) and metabolites dysregulated (feces and serum) in DJ-1 deficient in young adult mice.

## Results

### Gut microbiome dysbiosis (dysregulation of intestinal bacterial community signatures) in young DJ-1^−/−^ mice

Recent studies in sporadic PD patients have described the potential link with gut microbial abundance and Parkinson’s pathogenesis^28,29,31^. Further findings suggested that the brain-gut axis interactions are controlled by the gut microbiome through immunological, neuroendocrine and direct neural mechanisms, respectively^19,39^. Therefore, a clear understanding of the microbiota-gut-brain axis interaction could bring new insights in the pathophysiology of PD and allow an earlier diagnosis with a focus on peripheral biomarkers within the enteric nervous system^41^. However, if the DJ-1 deficiency has any potential effects on the gut microbiome is not known yet. To understand this process in more detail, we used the 16S rRNA sequencing method and characterized the gut microbiome from DJ-1^−/−^ and control littermate wild-type (WT or DJ-1^+/+^) fecal samples from 4 months old animals. Data analysis of 16S rRNA sequencing reads were performed using the MEGAN-CE microbiome analyzer software ^42^ as well as MicrobiomeAnalyst tool^43^.

**Figure 1:**
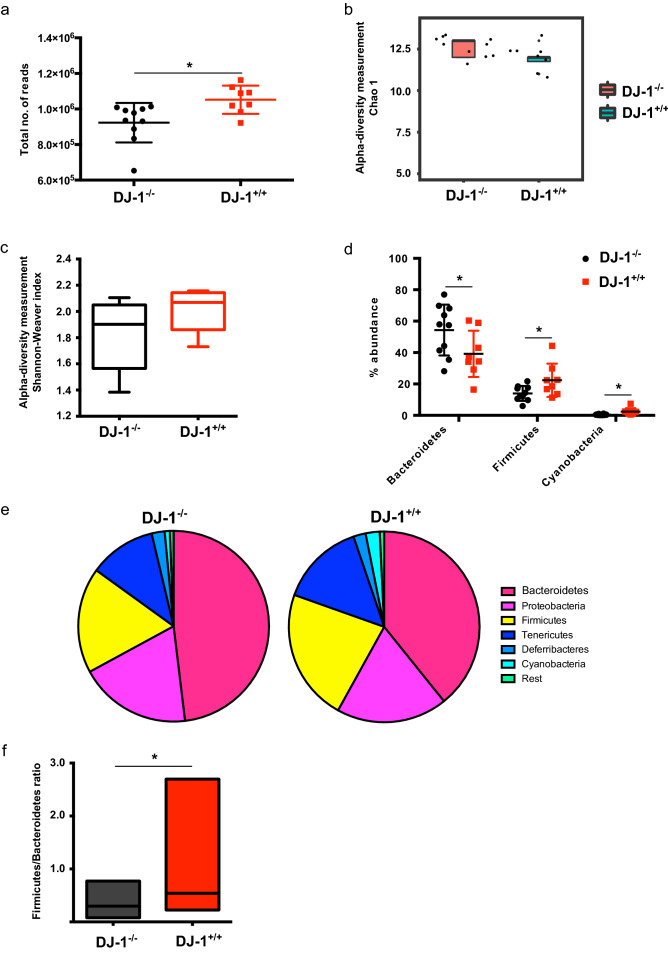
Gut dysbiosis is prevalent in 4 month-old DJ-1^−/−^ mice. **(a)** Total number of sequences reads obtained from sequencing of colon fecal samples from DJ-1^+/+^ (n = 8) and DJ-1^−/−^ (n = 10) mice. **(b, c)** Measurement of alpha diversities (Chao1 and Shannon–Weaver index) for DJ-1^+/+^ and DJ-1^−/−^ mice. **(d)** *Bacteroidetes, Firmicutes* and *Cyanobacteria* were significantly different between DJ-1^+/+^ and DJ-1^−/−^ mice. *represents the p value of ≤ 0.05 using Student’s unpaired t-test. **(e)** Bacterial abundance data (mean ± SD) presentation at phylum level in DJ-1^+/+^ and DJ-1^−/−^ mice. **(f)** Firmicutes/Bacteroidetes ratio in DJ-1^+/+^ and DJ-1^−/−^ mice, it was significantly reduced in DJ-1^−/−^ mice compared with DJ-1^+/+^. * represents the p value of ≤ 0.05 using Mann-Whitney U-test.

Total number of reads were significantly fewer in DJ-1^−/−^ samples compared with DJ-1^+/+^ samples (Fig. 1a). Our microbiome data at phylum level analysis suggested that 4 months old DJ-1^−/−^ mice tended to have higher alpha diversity Chao 1 and lower Shannon–Weaver index: species richness within a single microbial ecosystem^44^ of gut microbiome compared with the age-matched control DJ-1^+/+^, but it did not reach significance level (Fig. 1b,c). Similarly, beta diversity: diversity in microbial community between different environments^44^ tended to be reduced (UniFrac: both weighted and unweighted), a difference, however, again not reaching statistical significance. DJ-1^−/−^ mice have significantly higher abundance of *Bacteroidetes* (p = 0.05) and significantly less abundance of *Firmicutes* (p = 0.03) and *Cyanobacteria* (p = 0.04) compared with DJ-1^+/+^ (Fig. 1d). Further, when we mined the data for the overall composition of the gut bacterium at the phylum level, we found that indeed several bacterial phyla tended to be different, but the difference again did not reach statistical significance (Fig. 1e). As earlier studies suggested that *Firmicutes/Bacteroidetes* (F/B) could help to predict the functionality of the microbiome, hence, we calculated the F/B ratio and found that in DJ-1^−/−^ mice the F/B ratio was statistically significantly decreased (p = 0.03) when it was compared with DJ-1^+/+^ (Fig. 1f).

**Figure 2:**
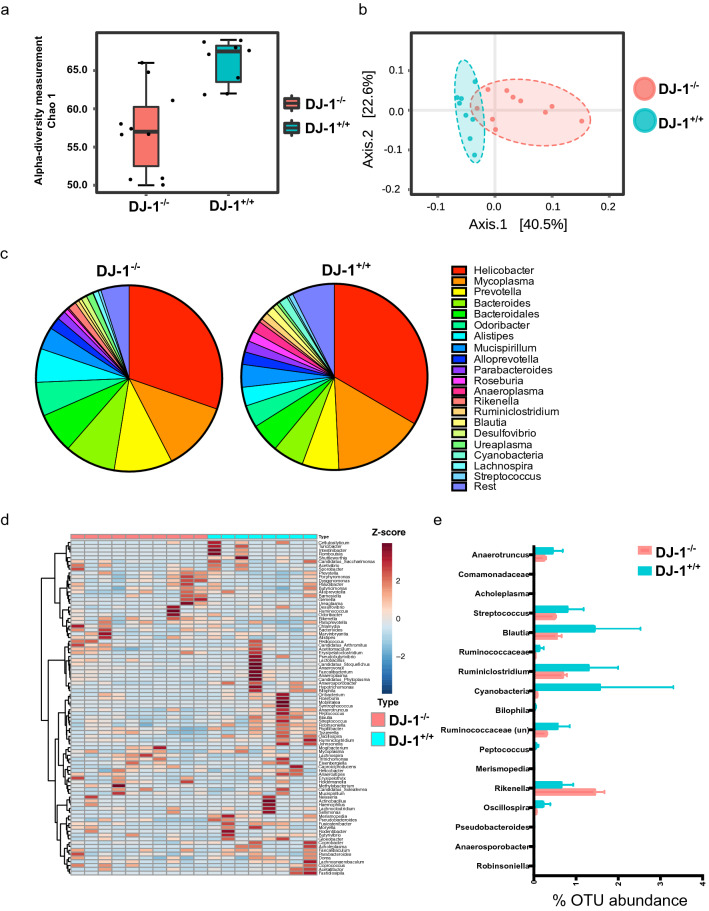
Increase in *Rikenella* and *Alistipes* bacteria in DJ-1^−/−^ mice compared with DJ-1^+/+^ at four months of age. **(a)** Measurement of alpha (Chao1) diversity in DJ-1^+/+^ and DJ-1^−/−^ mice. **(b)** Beta diversity (UniFrac) for WT (n = 8) and DJ-1^−/−^ (n = 10) mice. Both DJ-1^+/+^ and DJ-1^−/−^ mice clusters in a separate group. **(c)** A representative image of abundance of bacteria at genus levels in all DJ-1^+/+^ (n = 8) and DJ-1^−/−^ (n = 10) mice fecal samples. **(d)** Heat map represents the bacterial abundance in DJ-1^+/+^ and DJ-1^−/−^ mice in all the animals. The abundance of bacteria at genera level are represented in Z-score. **(e)** Average statistically significant (p value of < 0.05) bacterial abundance at genus level (only significantly abundant genera are shown) using Student’s unpaired t-test.

We further calculated the alpha and beta diversities at the genera level and found that both the alpha diversity (Chao1) was significantly reduced (p = 0.03) in DJ-1^−/−^ mice compared with DJ-1^+/+^ (Fig. 2a,b). Furthermore, analysis using Bray–Curtis principal component analysis (PCoA) with MEGAN-CE and UniFrac to differentiate between the two different genotypes (DJ-1^+/+^ and DJ-1^−/−^) and revealed that indeed, at a younger age both the animals were different in their bacteria abundance and clustering. Beta diversity was also calculated using MicrobiomeAnalyst tool^43^ (PERMANOVA). We observed that DJ-1^+/+^ and DJ-1^−/−^ were significantly (p value = 0.03; UniFrac) clustered into two different groups at the genera level (Fig. 2b). More than 1% bacterial genera were represented in a pie chart and clustering of bacterial genera were shown as heat map (Fig. 2c,d). In total 17 bacterial genera were significantly changed in the DJ-1^−/−^ animals (Fig. 2e). Most of the bacterial genera were significantly downregulated (*Anaerotruncus, Comamonadaceae, Acholeplasma, Streptococcus, Blautia, Ruminococcaceae, Cyanobacteria, Bilophila, Ruminococcaceae-uncultured, Peptococcus, Merismopedia, Oscillospira, Pseudobacteroides, Anaerosporobacter* and *Robinsoniella*), only the opportunistic symbionts *Rikenlla* (p = 0.01) and *Alistipes* (p = 0.07) were significantly upregulated (Fig. 2e). Although 16S rRNA sequencing is not specific enough to characterize the bacterium at species/strain level, however, it is sensitive enough to get an approximate idea which species could be present. Henceforth, we mined our 16S rRNA data and found that both unknown *Rikenella sp.* (p = 0.05), *Prevotella sp.* (p = 0.02) and *Alistipes timonensis* (p = 0.03) were significantly upregulated in DJ-1^−/−^ mice (Suppl. Figure 1a, b).

We next used the functional gene prediction profiling through Tax4Fun from our 16 s rRNA data set^45^ and output of these functional profile at KEGG metabolism level was visualized in SDP module of MicrobiomeAnalyst tool^43^. The association analysis was performed using Global test algorithm between the two groups (DJ-1^−/−^ and DJ-1^+/+^ mice). We observed that most of genes were predominantly associated with the pathways of amino acid, carbohydrate, energy, vitamins, cofactors and nucleotides metabolisms in WT and DJ-1^−/−^ mice (Suppl. Figure 2). However, these functional data are based on 16S rRNA sequencing data therefore, further analysis is needed based on genome-wide shot-gun methods of the bacterial genome to confirm these results. Overall, our data of the 16S rRNA bacterial sequencing analysis suggested that genetic deficiency of DJ-1 affects the bacterial composition and functions in the gut, even before these animals develop any disease phenotype.

### Increased inflammation in the feces and colonic tissues, compromised innate immune system in DJ-1^−/−^ mice

**Figure 3:**
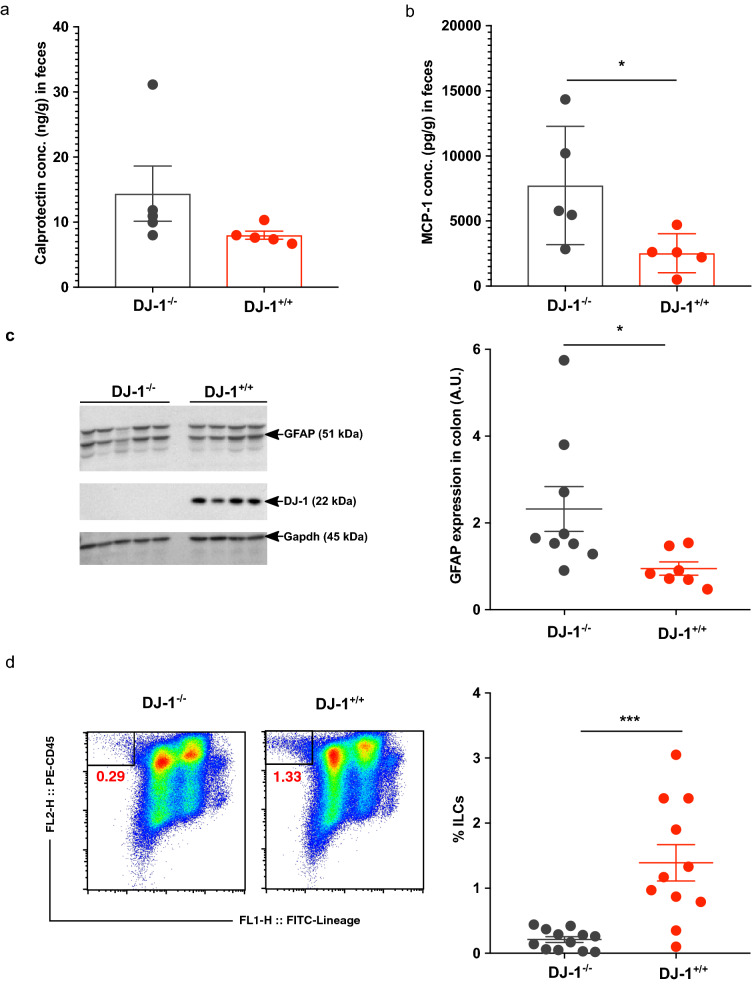
Increased inflammation and decreased ILCs in DJ-1^−/−^ mice. **(a)** Calprotectin level (mean ± SEM) measured by ELISA in DJ-1^+/+^ and DJ-1^−/−^ mice fecal samples (n = 5). **(b)** DJ-1^−/−^ mice have significantly higher production of the MCP-1 (mean ± SEM) cytokine measurement from the feces. * represents the p value of < 0.05 using Student’s unpaired t-test. **(c)** Original Immunoblots from GFAP/Gapdh expression and quantification of the protein expression. Images used in the manuscript were cropped from the original blots which are shown in Suppl. Figure 7 and contrast was adjusted to make the image uniform. Bar diagram (mean ± SEM) shows the statistical significantly higher expression of GFAP in the colon tissues of the DJ-1^−/−^ mice compared to control DJ-1^+/+^ mice (n = 7–9) from two different cohorts. **(d)** Left hand side figure shows representative FACS plots from DJ-1^+/+^ and DJ-1^−/−^ mice spleen samples. X-axis represents Lineage markers of lymphoid cells (CD3, CD5, CD3, CD11b, CD11c, F4/80, Gr-1, B220 or CD19, and Ter119) and y-axis represents lymphoid marker CD45. Right hand side Bar diagram (mean ± SEM) shows significantly lower in DJ-1^−/−^ (n = 12) compared with DJ-1^+/+^ (n = 11) from three different cohorts. *represents the p value of < 0.05 using Student’s unpaired t-test.

A dysregulated immune system could lead to inflammation and increased permeability of the gut^46,47^. Thus, we further characterized the inflammatory protein calprotectin, in the feces. Due to leukocytes shedding in the intestinal lumen, pro-inflammatory proteins such as calprotectin (S100A8/S100A9) can be detected and measured in the stool by ELISA^48^. The concentration of calprotectin is directly proportional to the intensity of the neutrophil infiltrate in the gut mucosa. Calprotectin is released from neutrophils, monocytes, macrophages and epithelial cells in the case of the GIT^48^. PD patients have a higher expression of inflammatory fecal calprotectin and zonulin (marker of increased gut permeability) proteins^47^. Further, another study also suggested enhanced inflammatory response in PD patients^49^. To validate whether increased gut dysbiosis in DJ-1^−/−^ mice is associated with higher inflammation in the gut or not, we measured the fecal calprotectin levels. In DJ-1^−/−^ mice calprotectin appeared to be higher than in WT, a difference, however, not reaching statistical significance (Fig. 3a). Moreover, we also measured other inflammatory cytokines in the feces and found that the monocyte chemotactic protein-1 (MCP-1) was significantly upregulated in DJ-1^−/−^ mice compared with control DJ-1^+/+^ mice (p = 0.03) (Fig. 3b). Other pro-inflammatory cytokines (IL-12p70, TNF-α, IL-17A, IFN-γ, IL-23 and IL-6) tended to be upregulated in the feces from DJ-1^−/−^ compared with DJ-1^+/+^ mice, however, did not reach statistical significance (Suppl. Figure 3). The cytokines GM-CSF, IFN-β and IL-27 were significantly down regulated in DJ-1^−/−^ mice compared to DJ-1^+/+^ (Supp. Figure 3). Additionally, previous studied suggested that pro-inflammatory cytokines are able to increase glial fibrillary acidic protein (GFAP) expression in enteric glia^50^ and PD patients have also enhanced inflammation ^49^, therefore, we explored the quantification of GFAP from the colon tissue. Our immunoblotting data suggested that DJ-1^−/−^ colon has significantly higher GFAP protein (p = 0.04) compared with DJ-1^+/+^ control mice and immunofluorescence staining highlight the presence of GFAP in enteric neurons (Fig. 3c and Suppl. Figure 4).

**Figure 4:**
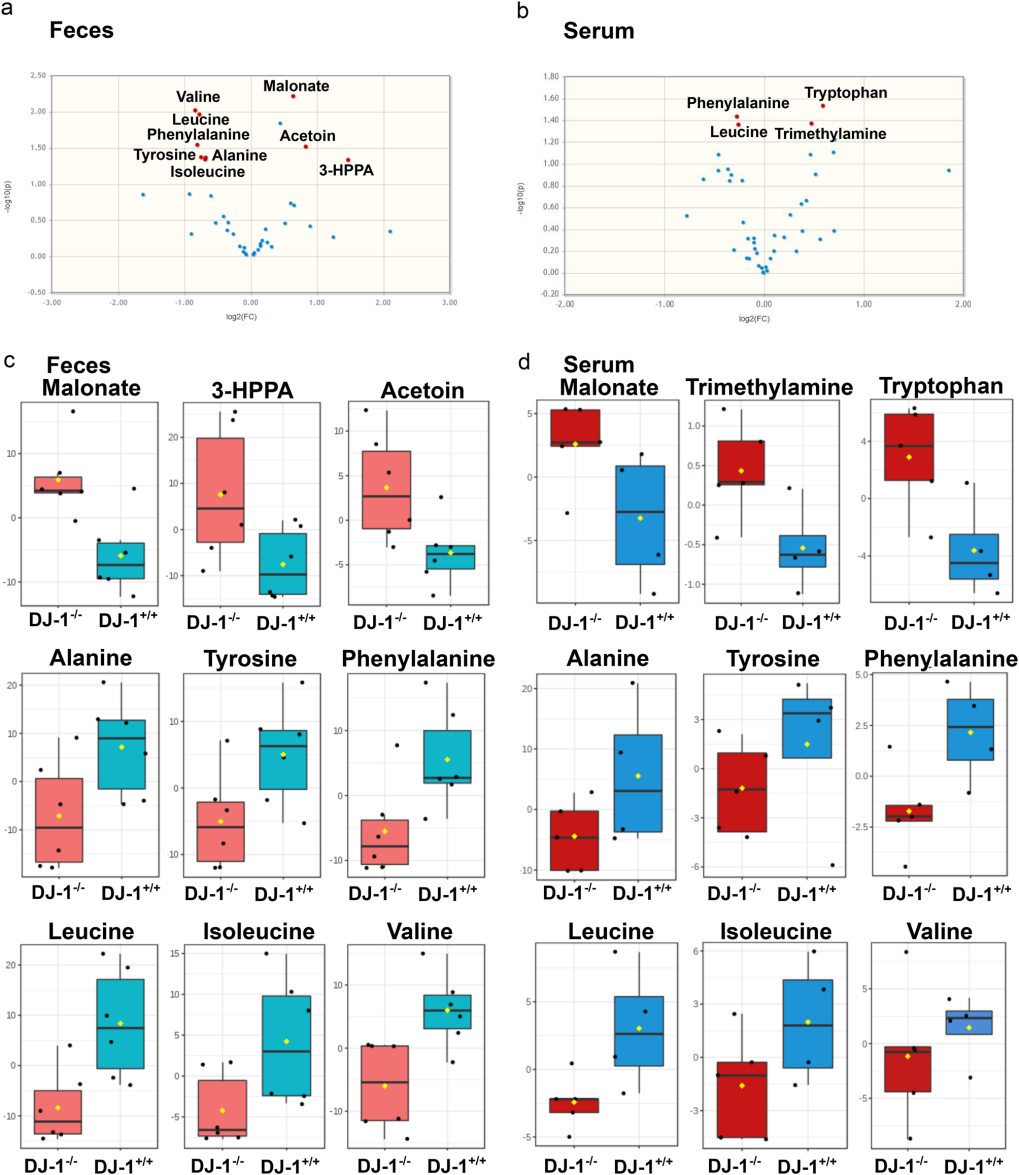
Reduced amino acids and increased SCFAs in feces from DJ-1^−/−^ mice feces and serum. **(a, b)** Volcano plot analysis of quantitative metabolite data (feces and serum) from ^1^H-NMR spectroscopy. **(c)** A total of nine metabolites (Malonate, 3-HPPA, Acetoin, Alanine, Tyrosine, Phenylalanine, Leucine, Isoleucine and Valine) showed p-values < 0.05 and fold changes > 1.5 between the two groups with Valine (high in DJ-1^+/+^) and Malonate (high in DJ-1^−/−^) being the most significant ones in feces. **(d)** A total of four metabolites (Phenylalanine, Trimethylamine, Tryptophan and Leucine) showed p-values < 0.05 and fold changes > 1.2 between the two groups with Leucine (high in DJ-1^+/+^) and Tryptophan (high in DJ-1^−/−^) being the most significant ones in serum.

Unwanted activation of the innate immune system from small intestinal bacterial overgrowth leads to increased intestinal permeability and may cause systemic inflammation^41^. In the same perspective, unwanted activation of enteric neurons and enteric glial cells could follow the initiation of α-Syn accumulation and misfolding in the gut and the brain^51^. In addition to the innate immune system, the adaptive immune system could also be affected and modified by bacterial proteins cross-reacting with host antigens which could lead to severe inflammation of host tissues^52^. Elevated α-Syn expression impairs innate immune cells function^53^. Our previous studies suggested that DJ-1^−/−^ mice have less induced regulatory T cells (Tregs)^16^. We speculated that the defect could be in the adaptive immune cell development or functions in the DJ-1^−/−^ mice. However, involvement of innate lymphoid cells (ILCs) in the pathogenesis of PD has not been described yet. To delineate the role of ILCs, we first characterized these cells using Flow cytometry and found that CD45^+^Lineage^−^ (CD3, CD5, CD3, CD11b, CD11c, F4/80, Gr-1, B220 or CD19, and Ter119) were significantly less abundant in DJ-1^−/−^ mice compared with WT control littermates in the spleen (Fig. 3d).

### Bacterial metabolites in the feces and serum of DJ-1^−/−^ mice are dysregulated

Our results suggested that DJ-1^−/−^ mice suffer from gut dysbiosis and as a result could have differences in their fecal metabolite production resulting in changed physiology and disease outcome. Several previous studies have identified that the gut bacteria can control different metabolites production which are involved in neurodegenerative diseases^19,54–56^. Therefore, we used proton nuclear magnetic resonance (^1^H-NMR) spectroscopy based metabolomics for the identification and quantification of fecal metabolites as described elsewhere in detail^57,58^. The detected compounds cover a comprehensive range of metabolite classes such as amino acids, SCFAs, phenols, amines, carbohydrates, purines, alcohols, and others.

Our extraction procedure yielded very rich and high-quality ^1^H-NMR spectra with final TSP linewidths < 1 Hz. A total of 40 metabolites could be annotated and quantified in all samples, mainly amino acids, short chain fatty acids, carbohydrates, and nucleotides. The clustering and non-clustering of all metabolites is shown in the heatmap (Supp. Figure 5). Aside from known metabolites we identified in half of the samples high levels of 3-(3-Hydroxyphenyl)propanoic acid (3-HPPA) (CAS 621-54-5), a phenol derivative which has been shown to be able to readily cross the gut epithelium^59^ into the blood and brain^60^ (Fig. 4; Supp. Figure 5). 3-HPPA has been shown to be formed mainly by *Clostridium, Escherichia* and *Eubacteria* species^61,62^.

**Figure 5:**
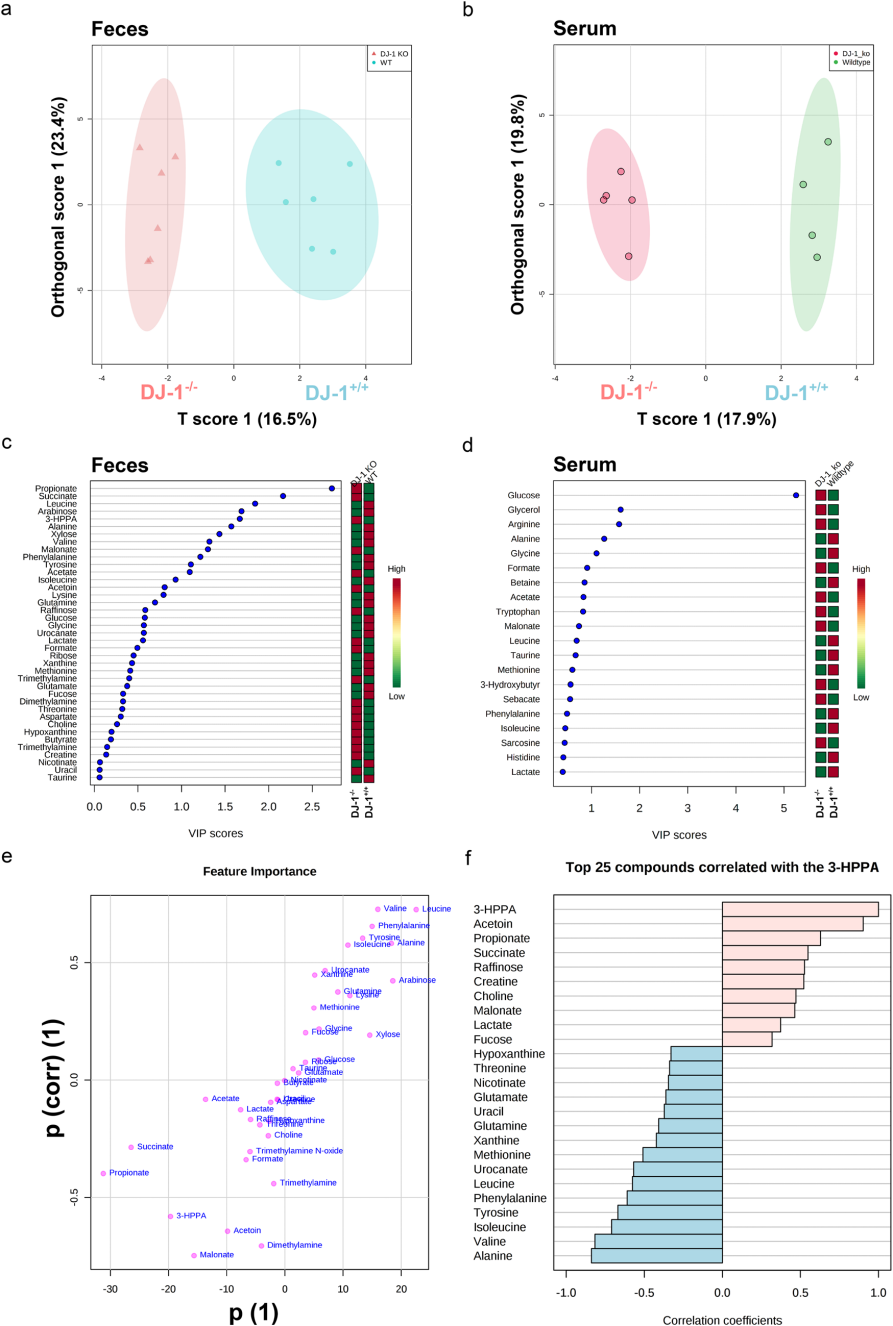
DJ-1^−/−^ feces metabolites differently clusters and have difference in metabolite production than the DJ-1^+/+^ feces. **(a, b)** Orthogonal partial least squares discriminant analysis (oPLS-DA) with the corresponding s-plot clearly separates DJ-1^+/+^ and DJ-1^−/−^ groups in feces and serum samples. **(c, d)** Partial least squares discriminant analysis PLS-DA with VIP scores for the top 20 metabolites in feces and serum samples. **(e)** SCFAs and amino acids being the most discriminating metabolites, indicating a general switch from protein towards fibre degrading strains in DJ-1^−/−^ feces. **(f)** The pattern hunter of the newly in feces identified phenolic metabolite 3-(3-Hydroxyphenyl) propanoic acid.

Using a classical volcano plot analysis with a fold change threshold of 1.5 and student’s unpaired t-test we identified 9 metabolites as highly discriminating the two groups (Fig. 4a) with malonate (p = 0.006) being top for DJ-1^−/−^ and valine (p = 0.008) for DJ-1^+/+^ in the feces (Fig. 4c). Similar analysis was performed for serum metabolites and we found that tryptophan (p = 0.02) phenylalanine (p = 0.03), leucine (p = 0.04), and trimethylamine (p = 0.04) were significantly different between DJ-1^−/−^ and DJ-1^+/+^ (Fig. 4b,d). Serum malonate (p = 0.08) tended to be likewise higher in feces in DJ-1^−/−^ compared with DJ-1^+/+^, however did not reach a significant level (Fig. 4d). Other serum metabolites such as alanine, tyrosine and isoleucine were also tended to be reduced (comparably like feces) in DJ-1^−/−^ mice compared with DJ-1^+/+^, however, also not reaching the significant threshold of p < 0.05 (Fig. 4d). Further multivariate statistics using orthogonal T-score (Partial Least Squares Discriminant Analysis: oPLS-DA) was employed to identify the similarity between DJ-1^−/−^ and DJ-1^+/+^ in feces and serum samples respectively (Fig. 5a,b, Suppl. Figure 5). We applied variable importance of projection (VIP) PLS-DA analysis to distinguish correlation patterns among all different metabolites (Fig. 5c,d). Our data analysis suggested that mostly SCFAs, amino acids, carbohydrates and aromatic compounds with VIP-scores > 1 were present in feces, however, in serum, it was somewhat less distinguished (Fig. 5c,d). Additionally, we found a strong separation of both groups and the corresponding s-plot demonstrates that SCFAs and amino acids were the most discriminating metabolites for clustering in feces samples (Fig. 5e). Interestingly the high abundance of SCFAs in DJ-1^−/−^ showed a high correlation with the newly identified metabolite 3-HPPA in feces (Fig. 5f and Supp. Figure 6). Thus, overall data suggested that DJ-1^−/−^ mice have impaired protein degradation—low levels of amino acids while fiber digestion was enhanced—high SCFAs production and could potentially trigger immune response and gut epithelial dysbiosis.

**Figure 6:**
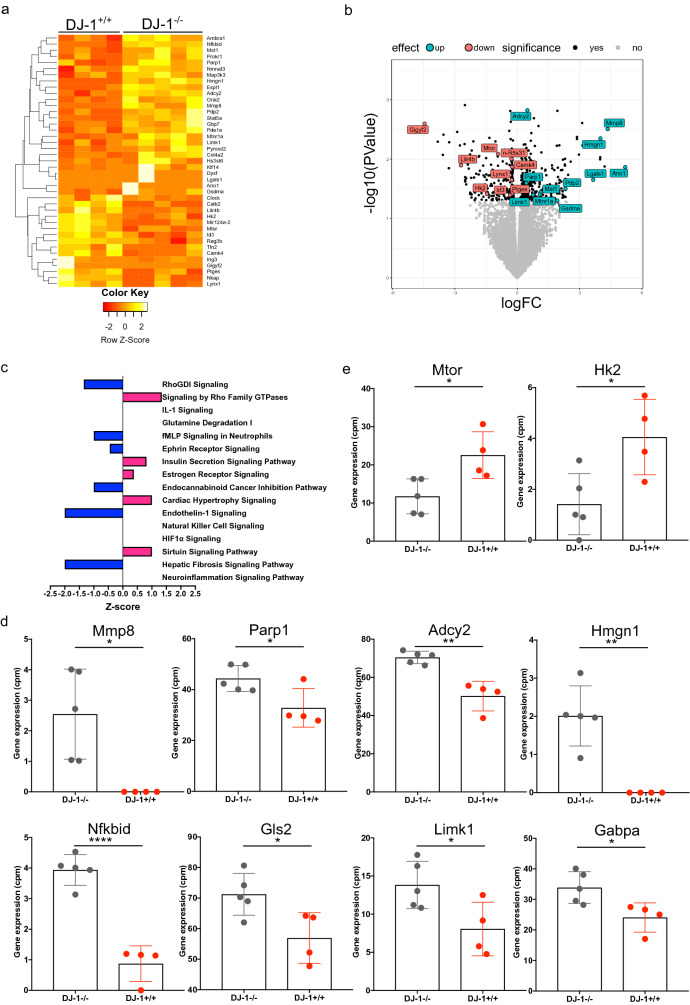
RNAs-seq analysis from mid-brain containing *SNpc* region. **(a)** Heatmap of differentially expressed genes (selected related with inflammation) in DJ-1^−/−^ and DJ-1^+/+^ mice. Color key showed the positive and negative z-scores. **(b)** Volcano plots of differentially up and down regulated genes. Only selected genes which are involved in inflammation were shown. Green color represented downregulated while Red color represented upregulated genes. Grey dots showed no significant change while black dots showed significant change in gene expression. **(c)** IPA analysis of differentially regulated pathways in DJ-1^−/−^ mice. **(d, e)** Gene expression (count per million; cpm) data for selected inflammatory genes. *(p ≤ 0.05), **(p ≤ 0.001) and ****(p ≤ 0.00001) respresent the p value significance based on Student’s unpaired t-test.

### Increased inflammatory genes related to PD in DJ-1^−/−^ mice midbrain region including SNpc

DJ-1^−/−^ mice have a dysbiotic microbiome composition and gut inflammation, but there was no evidence that DJ-1^−/−^ mice also develop neuro-inflammation at younger age at molecular level as previous studies only focussed on behaviour functional tests^7^. Earlier reports suggested that DJ-1^−/−^ mice do not exhibit any gross neuronal abnormalities, motor deficit (loss in DA neurons) and do not develop any PD like pathology even at older age unless they are challenged with amphetamine or MPTP^7^. To decipher whether microbiota-derived metabolites could be involved in triggering inflammation in the brain albeit at molecular level is not clear. Thus, we performed RNA-sequencing (RNA-seq) on the mid brain region containing *SNpc*. We found that total 529 genes were dysregulated (p value < 0.05). Out of 529 genes 275 genes were upregulated and 254 genes were downregulated (Suppl. Table 1). A few of the significantly dysregulated genes (both up and downregulated) were shown in heatmaps and volcano plots (Fig. 6a,b). Further, we performed the knowledge-based ingenuity pathway analysis (IPA) tool to identify the common pathways which could be dysregulated in the brain. We found that sirtuin signalling pathway^63^, cardiac hypertrophy signalling, estrogen receptor signalling, insulin secretion signalling^64^ and signalling by Rho Family GTPases were upregulated in DJ-1^−/−^ mice while hepatic fibrosis signaling pathway, endothelin-1 signalling, ephrin receptor signalling, fMLP signalling in neutrophils were down regulated based on Z-score (Fig. 6c). No change in Z-score was observed for neuro-inflammation signalling, Hypoxia-inducible factor-1 α (HIF-1α) signalling, Natural Killer cell signalling, Glutamine degradation I and IL-1 signalling (Fig. 6c). Further, individual gene expression analysis suggested that certain genes involved in PD inflammation were upregulated in DJ-1^−/−^ mice. The Poly(ADP-ribose) polymerase family, member 1 (Parp1) (p = 0.03) and Matrix metallopeptidase-8 (Mmp-8) (p = 0.01) genes which are involved in microglia and astroycytes inflammation^65–67^ were significantly upregulated in DJ-1^−/−^ mice in the mid brain regions (Fig. 6d). Adenylate cyclase 2 (Adcy2) which is involved in dopaminergic signalling^68^, IL-1 and several other pathways was also upregulated (p = 0.001) in DJ-1^−/−^ mice. High mobility group nucleosomal binding domain 1 (Hmgn1) is a transcription factor and it is involved in T helper type 1 inflammation^69^ and was also upregulated (p = 0.001) in DJ-1^−/−^ mice. Few other genes such as Nuclear factor of kappa light polypeptide gene enhancer in B cells inhibitor, delta (Nfkbid) (p = 0.0001), GA repeat binding protein, alpha (Gabpa) (p = 0.02), glutaminase 2 (Gls2) (p = 0.03), LIM-domain containing, protein kinase (Limk1) (p = 0.03) are linked with PD^70–72^ and were also upregulated in DJ-1^−/−^ compared with control DJ-1^+/+^ mice (Fig. 6d). The mechanistic target of rapamycin (serine/threonine kinase) (mTOR) (p = 0.02) and Hexokinase 2 (Hk2) (p = 0.02) genes were significantly downregulated in DJ-1^−/−^ mice, which are also involved in PD pathology in patients and animal models^64,73,74^ (Fig. 6e). Furthermore, Hk2, Mmp8 and mTOR also involved in HIF-1α signalling pathways, Adcy2 and Nfkbid predicted to be participate in IL-1 signaling and Gapba, Parp1 and mTOR are involved in sirtuin signaling pathways (Suppl. Table 2). Thus, RNA-seq data suggested that there could be inflammatory gene signatures in the mid brain of DJ-1^−/−^ mice.

## Discussion

Over the past years, our understanding of human-associated microorganisms has vastly been improved beyond that of a certain species toward an appreciation of the diverse and niche-specialized microbial communities that develop in the human host^75^. Our GIT especially the colon contains a largest reservoir of different microorganisms such as bacteria, viruses, fungi etc. interacting with the host epithelial and immune cells. These microorganisms orchestrate the primary and secondary metabolism on the gut mucosal surface and influence the other body organs, mucosal and hematopoietic immune functions^75^. Thus, it is not surprising that modulation in the composition and function of the gut bacteria has been linked with several chronic diseases including gastrointestinal inflammation, metabolic disorders as well as cardiovascular and neurological diseases^41,76–78^. Recent discoveries in the field of the gut microbiome and their role in the neurodegeneration in patients and animal models highlights an important link among each other^79^. As most of the common neurodegenerative diseases such as Alzheimer’s disease and PD occur late in life^40,80^ biomarkers allowing early diagnosis would be invaluable.

### Bacterial composition and dysbiosis

Animal models are useful tools to understand the pathophysiological functions of disease as these models allow us to manipulate the course of the disease^81^. To understand the impact of the gut microbiome in the pathophysiology of PD in early life prior to development of any phenotype or symptoms, we used DJ-1^−/−^ young adult mice. The DJ-1 mutation has been found in 1–2% early-onset recessive PD and PD patients containing DJ-1 mutation are normally responsive to levodopa^8^. We found that two novel bacteria such as *Alistipes* and *Rikenella* genera were highly abundant in DJ-1^−/−^ mice compared with WT control littermate animals even at 4 months age. Both the alpha and beta diversity analysis suggested that both genotypes of animals have a distinct microbiota diversity and community. Gorecki et al. did not observe differences in Thy-1 aSyn Tg mice compared with WT mice at early age (2 months)^82^. We also have a similar finding as described in Gorecki et al. in our a-Syn (BAC-hSNCA TG) mouse model^83^. We did not find any major difference at 6 months age in BAC-hSNCA TG mice model, either. Thus, the results observed in the present study could be distinct from the pathology associated with PD. Furthermore, *Alistipes* and *Rikenella sp*. have been described earlier to be involved in the inflammatory bowel disease as well as colon cancer development^84^. In humans (colorectal cancer patients) a number of Bacteroides and Parabacteroides species, along with *Alistipes putredinis, Bilophila wadsworthia, Lachnospiraceae bacterium* and *Escherichia coli* were enriched in carcinoma samples compared with both healthy and advanced adenoma samples^85^. *Alistipes* and *Ruminococcus* were shown to be positively correlated with TNF-α production after anti-IL-10R/CpG oligonucleotide immunotherapy in C57BL/6 mice suffering from MC38 colon carcinoma^86^.

Chondroitin sulfate has been shown to increase the abundance of *Rikenella*, a genus of sulphate reduced bacteria, but did not significantly change the abundance of *Akkermansia muciniphila*^87^. Surprisingly, *Rikenella* was eliminated by cephalosporin whereas *Rikenella* blossomed on exposure to berberine. However, *A. muciniphila* was eliminated by this antibiotic compound. Cephalosporin significantly reduced colonic mucus lesions and delayed the early pathogenesis of dementia, steatohepatitis and atherosclerosis. Berberine significantly aggravated colonic mucus lesions and enhanced multi-systemic pathogenesis^87^. Thus, *Rikenella* could be involved in sulphate reduction in DJ-1^−/−^ mice and could be a triggering factor to induce colonic mucus lesions and PD disease progression. *Alistipes* and *Rikenella* belong to *Rikenellaceae* family which is positively correlated with PD pathology based on the Unified Parkinson’s Disease Rating Scale (UPDRS)-III in PD patients^88^. Similarly, like DJ-1^−/−^ mice, both the bacterial strains are higher in BAC-hSNCA TG mice PD model at 12 months in the cecum and colon, however no change occurred at a younger age (6 months) as described earlier^83^. This early life bacterial dysbiosis could be linked with PD pathogenesis, however, these data are not longitudinal, therefore, it is not possible to determine whether dysbiosis is a cause or consequence of neuroinflammation and PD pathogenesis. Nonetheless, our data highlights the importance of gut bacterial genera/species in sporadic PD patients and use of animal model to decipher the disease mechanisms.

### Intestinal inflammation regulation by inflammatory bacteria and immune cells

Intestinal inflammation has been linked with gut permeability and induction of immune response as well as function of gut neurons^19,75^. DJ-1^−/−^ mice challenged with LPS have increased production of inflammatory cytokines IFN-γ and interferon-inducible T-cell α chemoattractant from microglial cells^89^. These microglial cytokines appeared to be involved in loss of *SNpc* compared with WT mice^89^. These results suggested that interaction of a genetic defect with inflammatory mediators could participate in the development of PD. Our data also suggested that DJ-1^−/−^ mice suffer from higher fecal inflammation as well as neuronal inflammation (GFAP), which could be linked with neurodegeneration. However, the DJ-1^−/−^ mice do not develop any apparent phenotype in unchallenged conditions.

It is noteworthy that the current study was performed at early stage before any neurological symptom develops. Furthermore, brain transcriptomics data highlights that there could a low-grade inflammation in the brain of DJ-1^−/−^ mice, which may be involved in microglial activation. Microglial priming is caused by aging and neurodegenerative diseases. Parp1 is a chromatin-associated enzyme that participates in processes such as transcription and DNA repair through the regulation of chromatin structure^65^. The important role for parp1 enzymatic activity is involved in the expression of inflammatory cytokines in glial cells of central nervous system inflammation^65^. Furthermore, the pathological role of Mmps family members has been described in PD patients and animal models^66,67,90,91^. Treatment of Mmp-8 inhibitor on the brain of aged normal and leucine-rich repeat kinase 2 (LRRK2) G2019S PD model mice systemically stimulated with LPS suggested that Iba-1 positive microglia and GFAP-positive astrocytes were decreased which were activated by LPS in aged normal and PD mice model^66^. Thus, it is plausible that both Parp1 and Mmp8 could potentially be involved in the microglial activation and inflammation in DJ-1^−/−^ mice as well. The several genes are involved in different signaling pathways, however, how they are intersecting and inducing the inflammation to the mid brain region including the SNpc is yet to be determined.

Recent studies suggested that DJ-1C57^−/−^ with a different genetic background (backcrossed 14 generations with C57BL/6 mice), is more penetrant in disease phenotype development, as these animals develop the phenotype within 3 months of age and progress to by 12 months^14^. The exact mechanism is unknown, but it could possibly be correlated with the genetic background composition in an interplay with the gut microbiome development. However, further studies, in particularly also of the microbiome, are warranted to understand the disease penetration in DJ-1C57^−/−^ mice and DJ-1^−/−^ animals. DJ-1^−/−^ mice have no appreciable change in α-Syn expression in the colon (Suppl. Figure 4). Henceforth, absence of DJ-1 protein may trigger mechanisms which are not dependent upon α-Synucleinopathy. Interestingly, we found that ILCs are reduced in DJ-1^−/−^ mice. Thus, innate immunity is severely compromised such as high abundance of *Alistipes* and *Rikenella*. Previous studies suggested that *Alistipes sp.* thrived in the absence of lipocalin 2 and in IL-10^−/−^ mice and induced intestinal inflammation and cancer progression in those animals^84^. Moreover, NOD2 or RIP2 deficiency resulted in a pro-inflammatory microenvironment that enhanced epithelial dysplasia following chemical injury and causes gut dysbiosis probably due to higher abundance of *Rikenella* bacterium in these mice^92^. Interestingly, *Rikenella* was also shown to flourish in MyD88-deficient mice as these mice also lack a suitable innate immune system^93^. Gender-specific differences found in the immune system and gut microbiome composition in males and females apparently fosters the expansion of *Alistipes, Rikenella* and *Porphyromonadaceae* in the absence of innate immune defence mechanism in male mice^94^. These bacterial groups were linked with induction of weight loss, inflammation and DNA damage upon transfer of the male microbiota to germ-free female recipients^94^. This study points out that it could be the case as DJ-1^−/−^ mice have lower numbers of ILCs and increased inflammation as well as higher abundance of *Alistipes* and *Rikenella*.

### Dysregulation of metabolites and neurodegeneration

The gut microbiome is a complex biological system and exhibits various tasks for its host, including digestion, degradation of macromolecules, vitamin production and educating of the host innate and adaptive immune system^95^. The gut microbiome composition also affects the health of the host via modulating the concentrations of bacterial metabolites including SCFAs, trimethylamine N-oxide (TMAO) and other metabolites, respectively^78^. Bacterial metabolites appear to have diverse effects on metabolism and immune response and are considered as biomarkers for disease and risk factor, whereas bacterial components cause an innate inflammatory response^78^. Our metabolite data (feces and serum) suggests that amino acids including valine, leucine, phenylalanine, alanine, tyrosine and isoleucine were downregulated, whereas SCFAs including malonate, dimethylamine, trimethylamine and acetoin were upregulated in DJ-1^−/−^ mice. Defects in mitochondrial energy metabolism have been involved in the pathology of several neurodegenerative diseases^96^. Furthermore, metabolites generated during the bacterial metabolism and oxidation of the neurotransmitter DA are considered to damage the neurons of the basal ganglia^96^. Furthermore, mitochondrial respiratory complex II (CII) is a protein complex located in the inner membrane of mitochondria and it forms part of the electron transport chain and is involved in succinate signalling and reactive oxygen species (ROS)^97^. Malonate is a competitive inhibitor of the CII and reduces the cellular respiration, whereas succinate rather drives the CII activity in macrophages^98,99^. Infusion of malonate into the striatum of mice or rats produced degeneration of DA nerve terminals in striatum and malonate induces a substantial increase in DA efflux in awake, behaving mice as quantified by in vivo microdialysis^96^. Decrease of the SCFAs butyrate and propionate suggest loss of lactate utilizing bacterial strains^100^. In PD patients fecal SCFAs were reduced compared with healthy controls^31^. However, in DJ-1^−/−^ mice, SCFAs were similar to those of WT mice. Thus, metabolic stress induced by malonate and trimethylamine could be involved in the neurodegenerative process slowly in DJ-1^−/−^ mice potentially generated from bacterial metabolism or colon tissue.

The vast majority of amino acids in the intestines are derived from the metabolism of ingested dietary proteins, host tissue proteins or the conversion of other nitrogenous substances, whereas a small amount of amino acids is de novo synthesized by the gut bacteria^101^. Amino acids, such as glutamine function as a double-edged sword for gut health as they can sponsor the expression of pathogenic virulence genes as well as protect against disease^101^. Most of the amino acids including valine, leucine, isoleucine and alanine tended to be downregulated in the feces as well as serum of DJ-1^−/−^mice. Our results corroborate findings on PD patients cerebrospinal fluid (CSF) analysis for the amino acids including valine, leucine and isoleucine^102^. Thus, PD patients might suffer from dysfunction of the transport of neutral and basic amino acids across the blood–brain barrier^102^. Additionally, changes in amino acid metabolism in plasma/CSF of PD patients highlight the role of altered amino acid metabolism in PD pathology^103^. Decreased amino acid concentrations could be involved in loss of microbial proteases/peptide catabolism^100^. Apparently, there is a switch from protein to fibre utilization under DJ-1 deficient conditions^104^. PD patients who are not treated with levodopa or with dopamine agonists were reported to have higher CSF tyrosine and phenylalanine levels than those not treated with these drugs and also than controls^102^. In this study, we found a trend towards less tyrosine and phenylalanine in the feces and serum of DJ-1^−/−^ mice compared with DJ-1^+/+^ controls. Among the significantly identified amino acids within our tests, tyrosine and phenylalanine could be the most important as they are involved in L-DOPA synthesis by tyrosine hydroxylase (TH), which is further converted into dopamine, norepinephrine (noradrenaline), and epinephrine (adrenaline)^105^. It is shown that 3-HPPA is formed through fermentation of tyrosine by Clostridium species^61,62^. Reduced levels of microbial tyrosine and phenylalanine point out at decreased production of dopamine precursors while at the same time enhanced levels of 3-HPPA which are able to cross into the brain might have a direct effect on dopamine synthesis e.g. as competitive inhibitor. Tryptophan tended to be higher in serum of DJ-1^−/−^ mice, which is also a precursor of serotonin. A recent study in DJ-1 deficient rat model suggested that at 4 months of age, DJ-1 KO rats also had a tendency of increased serotonin levels in dorsal striatal region and it is significantly increase in older rats (8 months)^106^. Thus, it appeared that balance of metabolites would be implicated in regulation of PD progression and pathology. However, further studies are warranted to understand the role of amino acid metabolism for gut bacteria in PD pathophysiology. Further, this study does have some limitations and does not explain whether change in the brain inflammatory gene transcripts could also be due to genetic deficiency of DJ-1 or exclusively due to change in the microbiome. A follow up study with GF animal or antibiotics treatment will pin-point whether low-grade brain inflammation arises from the microbiome and their metabolites or it is inherent due to DJ-1 mutation.

## Conclusions

Our study suggests that DJ-1^−/−^ mice present with gut dysbiosis, reduced innate lymphoid cells numbers or development, increased inflammation (colon, feces and brain) and dysregulated metabolites production already at an early disease stage (4 months) which could be toxic to colon tissues or neurons. Therefore, these disease markers could subsequently be explored for biomarker development in PD patients.

## Material and methods

### Animal breeding and ethical permission and materials collection

DJ-1^−/−^ mice were described earlier^7^ and obtained from Prof. Tak W Mak, Toronto, Canada. As described earlier, F1 progeny were backcrossed for seven generation to C57BL/6 mice and heterozygous animals (male and female) were used to set up the breeding to obtain homozygotes for the targeted DJ-1 allele. Genotypes of mice was performed using PCR (WT DJ-1 forward primer, TGC TGA AAC TCT GCC ATG TGA ACC; WT DJ-1 reverse primer, CCT GCT TGC CGA ATA TCA T; and Neo, AGG TGA CAC TGC CAG TTG CTA GTC). PCR conditions were used as follow: 95 °C for 30 s, 64 °C for 30 s, and 72 °C for 1 min (40 cycles). All the animals were kept in open cages in a standard environment mice facility. Both the DJ-1^+/+^ and DJ-1^−/−^, were kept in the same cage and animals were kept in 5–6 different cages to nullify the cage effect as mice are coprophagic in nature. Heterozygous mothers were mated to obtain DJ-1^−/−^ and DJ-1^+/+^ animals to minimize the effect of the maternal microbiome. Age matched 3–4 months old DJ-1^+/+^ and DJ-1^−/−^ were sacrificed using CO_2_/Isoflurane methods and colons (3–4 cm long piece from at the junction of caecum) and were collected in 4% paraformaldehyde (PFA) and snap frozen in liquid N_2_. Furthermore, the same cohort of animals was also used for the feces collection (feces excreta obtained from entire length of colon) for 16S rRNA microbiome analysis and stored at − 80 °C until use. The spleens were used for characterization of ILCs.

### Bacterial DNA isolation from the feces

Frozen feces were weighted (50 mg/sample) on dry ice and hammered to break into powder form and transferred into a 2.0 ml Eppendorf tube and kept on dry ice. Once all the samples were measured, all the weighted sample tubes were transferred to ice until the samples were thawed and 1.0 ml of lysis buffer from QIAamp Fast DNA Stool Mini Kit (Cat no. #51604; Qiagen, Germany) was added. All the procedures were followed as recommended by manufacturer’s guidelines for bacterial DNA isolation and DNA was dissolved in 100 µl instead of 200 µl DNA buffer.

### 16S rRNA sequencing

For 16S rRNA amplification, 12.5 ng of DNA was amplified using 0.2 μM of both forward primer (TCGTCGGCAGCGTCAGATGTGTATAAGAGACAGCCTACGGGNGGCWGCAG, Metabion) and reverse primer (GTCTCGTGGGCTCGGAGATGTGTATAAGAGACAGGACTACHVGGGTATCTAATCC, Metabion).

KAPA HiFi HotStart Ready Mix (KK2601; KAPABiosystems) was used for the PCR amplification. PCR was performed using a first denaturation of 95 °C for 3 min (min), followed by 25 cycles of amplification at 95 °C for 30 s, 55 °C for 30 s and 72 °C for 30 s, final elongation at 72 °C for 5 min and the amplified DNA was stored at 4 °C. DNA gel electrophoresis of all the samples was performed to verify the amplicon specificity.

Further, samples were then purified (Agencourt AMPure XP, Beckman Coulter) and PCR amplicons were indexed using Nextera XT index and KAPA HiFi HotStart ReadyMix. PCR was performed using a first denaturation of 95 °C for 3 min, followed by 8 cycles of amplification at 95 °C for 30 s, 55 °C for 30 s and 72 °C for 30, final elongation at 72 °C for 5 min. Samples purified were then validated using BioAnalyzer (Bioanalyzer DNA 1000, Agilent) and 4 nM of each library pooled using unique indices before sequencing on a MiSeq (Illumina) and paired 300-bp reads.

### Sequence analysis and statistics

Available sequence data was trimmed and filtered using SeqPurge ^107^. Trimming parameters demanded a minimum quality at 3′ end of q = 35 (parameter qcut = 35). Processed sequence data was aligned using MALT (version 0.3.8; https://ab.inf.uni-tuebingen.de/software/malt) against the 16S database SILVA SSU Ref Nr 99 (https://www.arb-silva.de/documentation/release-128/) and classified using NCBI taxonomy. Alignment was performed using semi-global alignment and a minimum sequence identity of 90% (parameter minPercentIdentity = 90). Further analysis and visualization were performed using MEtaGenome Analyzer-Community Edition (MEGAN-CE) version 6.14.2, built 23 Jan 2019^42^ as describer earlier^108^.

### Preparation of tissue for histological analysis

Colon samples prepared on ice were fixed for 24 h in 4% PFA, stored at 4 °C in 0.4% PFA for a maximum of 4 weeks prior embedding in paraffin. Fixed samples were then alcohol-dehydrated and embedded in paraffin. Samples were embedded in paraffin blocks using a tissue embedding station and stored at room temperature until use. Paraffin blocks containing colon tissues were cut into 7 μm thick sections using a microtome. Section were placed in 45 °C water bath for flattening, collected on a glass slide, dried in an incubator at 50 °C for 1–2 h and stored at room temperature.

### Immunohistochemistry (IHC) and immunofluorescence staining

First slides were deparaffinized using autostained program 6 for a run of 51 min and slides were kept in TBS until antigen retrieval step. Antigen retrieval was done using sodium citrate buffer (1 ×) and citric acid (1 ×) method. Slides were boiled in the citric acid + sodium citrate buffer for 5 min for three times (3 ×) and slides were allowed to cool down for 15 min in TBS. After cooling slides were blocked of endogenous peroxidase and incubated for 20 min at room temperature (RT) and washed quickly 3 × with TBS. Further slides were blocked for unspecific bindings using 5% normal goat serum in 0.3% Triton X-100 TBS and incubated for 1 h at RT on a slow rotation shaker. After incubation with unspecific binding, slides were washed for 3 × 3 min with TBS. Antibody staining was performed using α-Synuclein antibody (# 610786 BD Biosciences, Netherlands) for overnight (1:1000) dilution at 4 ∘C. Slides were washed next day with 0.025% Triton X-100 TBS for 3 × 5 min. Secondary antibody was used (1:1000 dilution) for 1 h at RT. Further slides were stained with ABC complex-DAB for detection of primary antibody staining. For immunofluorescence staining of GFAP, tissue sections were subjected to same treatment as IHC until primary staining with GFAP antibody (1:1000; #M0762, Dako, Demark) dilution at 4 ∘C for overnight. Slides were washed next day with 0.025% Triton X-100 TBS for 3 × 5 min. Secondary anti-Rabbit antibody raised in goat labelled with Alexaflour 488 was used (1:500 dilution) for 1 h at RT. The slides were mounted with ProLong Gold antifade reagent with DAPI (#P36931, Invitrogen, Germany). Microscopy was performed with an EVOS M7000 cell imaging system (Thermofisher).

### Tissue lysate preparation for WB

Colon tissues were weighted frozen and lysed with 10 volumes of RIPA buffer (50 mM Tris, 150 mM NaCl, 1.0% NP-40, 0.5% sodium deoxycholate, 0.1% SDS, pH 8.0) supplemented with protease inhibitor (Complete; Roche Diagnostics). Colon tissues were homogenized for 1 min using a disperser (T10 ULTRA-TURRAX; VWR) on ice. After the homogenization, samples were incubated for 30 min at 4 °C and spun for 20 min at 12,000 *g* at 4 ∘C. Proteins lysate supernatants were supplemented with 10% glycerol before long storage at − 80 °C. Protein concentration was determined using BCA method (#23225; Thermofisher, Germany).

### Immunodetection

Samples were prepared by diluting protein lysates in PAGE buffer (0.2 M glycine, 25 mM Tris, 1% SDS), followed by a denaturation at 95 °C for 10 min in loading buffer (80 mM Tris, 2% SDS, 5% 2-mercaptoethanol, 10% glycerol, 0.005% bromophenol blue, pH 6.8) and a short centrifugation 30 s at 400 *g*. Proteins were separated by electrophoresis using 12% SDS-PAGE gel. Gels containing proteins were washed for 5 min in transfer buffer (0.2 M glycine, 25 mM Tris, 10–20% methanol) and transferred to membranes equilibrated in transfer buffer. Transfer was performed for 90 min at 80 V at 4 °C on nitrocellulose membranes (88018, Life Technology). Immunoblot were washed 5 min in TBS buffer, fixed with 4% PFA for 1 h (only for a-Syn detection) and blocked using 5% non-fat milk (Slim Fast) in TBS. Membranes were then washed twice 5 min in TBST and incubated with the primary antibody over night at 4 °C (human and mouse a-syn: 610786 BD Biosciences; GAPDH: #5174, Cell Signaling and GFAP #MAB360, Merck Millipore, Germany). After incubation with the first antibody, membranes were washed four times (5 min each) with TBST. Membranes were then incubated for 75 min with the secondary antibody coupled to horseradish peroxidase (GE Healthcare). After four washing steps with TBST (5 min each), bands were visualized using the enhanced chemiluminescence method (ECL+; GE Healthcare). Light signal was detected using LI-COR Odyssey and were quantified using Odyssey software with standard setting. All the images have been provided (Suppl. Figure 6).

### Calprotectin ELISA and LEGENplex inflammatory cytokines

To measure calprotectin/MRP 8/14 in the fecal samples, S100A8/S100A9 ELISA kit rat/mice (#K6936, Immundiagnostik AG, Germany) as well as LEGENplex™ (#740150 or #740446, Biolegend, Germany) mouse inflammation panel (13-plex) were used according to the manufacture’s guidelines. Fecal samples were measured (weight between 50 ± 5.0 mg) and dissolved in 500 µl of extraction buffer supplied by the kit, mixed by vortexing and then centrifuged for 10 min at 3000 × *g*. Supernatant was taken and transferred to a new microcentrifuge tube and 100 µl of sample was used for measuring the protein for calprotectin, however, for LEGENDplex inflammatory panel 25 µl of sample was used respectively. The data were analyzed using 4 parameters algorithm and the concentration of calprotectin/inflammatory cytokines was normalized with feces weight and data are presented in ng/g or pg/g.

### Flow cytometry

Splenocytes from WT and DJ-1-deficient mice were characterized by using surface and intracellular staining with relevant antibodies. In brief, splenocytes were collected and used for surface staining for dump-FITC (channel) for various lineage markers—CD3, CD5, CD3, CD11b, CD11c, F4/80, Gr-1, B220 or CD19 and Ter119 and CD45-PE (eBioscience, Germany) for 30 min at room temperature. After incubation, antibodies labelled cells were washed with PBS. Cells were fixed with Foxp3 fixation/permeabilization buffer (eBioscience, Germany) for intracellular staining and incubated for 30 min. After incubation, cells were washed with 1 × permeabilization buffer, exposed to added intracellular monoclonal antibodies for RoRgt-PerCP-Cy5.5, Eomes-APC, Tbet-PerCP-Cy5.5 and GATA3-APC and incubated for an additional 45 min. Cells were washed again with permeabilization buffer and PBS was added to acquire the cells on a flow cytometer (FACS-calibur™ from Becton Dickinson; Heidelberg, Germany).

### Feces and serum samples preparation for the metabolite detection using ^1^H-NMR

For metabolite extraction, 50 mg of deep-frozen feces or 50 µL of serum samples were transferred into 2 mL AFA glass tubes (Covaris Inc, Woburn, USA) and mixed with 400 µL of ultrapure methanol and 800 µL of MTBE (solvent grade). The mixture was manually dispersed with a disposable plastic spatula, then vortexed and transferred to a focused ultrasonicator (Covaris E220evolution, Woburn, USA). Feces metabolites were extracted with a 5 min lasting ultrasonication program in a degassed water bath at 7 °C. After extraction, the metabolite suspension was separated into a polar and lipid phase by adding 400 µL of ultrapure water. In order to remove any remaining solids from the samples, the glass tubes were centrifuged for 5 min at 4000 rpm. 700 µL of each phase were then transferred to a fresh 1.5 mL Eppendorf cup. The polar phase was subjected to a 2nd centrifugation step for 5 min at 12,000 rpm and 600 µL of the supernatant were transferred to a new 1.5 mL Eppendorf cup and evaporated to dryness over night with a vacuum concentrator (Eppendorf Speedvac).

### ^1^H-NMR for metabolites and data analysis

For NMR analysis, the dried pellets (either from feces or serum) were resuspended with 60 µL of deuterated phosphate buffer (200 mM K2HPO4, 200 µM NaN3, pH 7.4) containing 1 mM of the internal standard TSP (trimethylsilylpropanoic acid). In order to allow for maximum dissolution, the plastic tubes were quickly sonicated and then centrifuged 5 min at 14,000 rpm. 50 µL of the supernatant were transferred with gel loading pipette tips into 1.7 mm NMR tubes (Bruker BioSpin, Karlsruhe, Germany) and a 96 well rack placed into the cooled (4° C) NMR autosampler.

Spectra were recorded on a 600 MHz ultra-shielded NMR spectrometer (Avance III, Bruker BioSpin GmbH) equipped with a triple resonance (1H, 13C, 31P) 1.7 mm room temperature probe at 298 K. For optimum water suppression and shim adjustment a quick simple ZG experiment was performed followed by a 1 h lasting CPMG (Carr-Purcell-Meiboom-Gill) experiment in order to suppress residual background signals from macromolecules such as bilirubin (time domain = 64 k points, sweep width = 20 ppm, 512 scans). The recorded free induction decays (FIDs) were fourier-transformed and spectra properly phase- and baseline corrected. Metabolite annotation and quantification was performed with ChenomX NMR Suite 8.3 and statistical analysis with MetaboAnalyst 4.0.

The phenol derivative 3-(3-hydroxyphenyl) propanoic acid 3-HPPA was identified by selective TOCSY experiments followed by purchasing possibly fitting reference standards of different phenols with substituted groups in the meta position. Reference spectra of those were recorded in the used feces extract phosphate buffer and by applying spiking experiments.

### RNA-seq for mid brain containing SNpc region

The mid brain containing SNpc region was isolated from freshly euthanized DJ-1^−/−^ and DJ-1^+/+^ mice (3–4 months age) and snap frozen on dry ice. Roughly 10–15 mg of tissue was used for total RNA isolation using Tissue lyzer II for 2 min at 30 Hz and mRNAeasy mini kit. Total RNA was used for library preparation, sequencing and data analysis as described earlier ^83^. Further, data mining was done using IPA software (Qiagen, Germany).

### Statistical analysis

MEGAN-CE (version 6.14.2, built 23 Jan 2019) and MicrobiomeAnalyst were used for data acquisition and analysis. GraphPad and Inkscape were used for the final figure preparation. One-way ANOVA, Mann-Whitney U-test or Student’s t-test was used for statistical analysis using GraphPad wherever it was appropriate and described in the figure legend. Data shown are either Means ± SD or SEM. P value ≤ 0.05 was considered significant. Metabolite concentrations from ^1^H-NMR analysis were exported as comma separated value spreadsheet file to MetaboAnalyst, normalized with PQN (probabilistic quantile normalization) and range scaled and then analyzed with student’s t-test, oPLSDA and VIP analysis.

### Ethics statement and approval

All the experiments were performed according to the EU Animals Scientific Procedures Act (2010/63/EU) and the German law for the welfare of the animals. All the procedure and methods were approved by the local government authorities (Regierungspräsidium, Tübingen according to §4 animal welfare act (permission granted on 20/07/2017 for the experiments) and Regiergungspräsidium, Tübingen/Stuttgart: (T195/20)) of the state of Baden-Württemberg, Germany.

### Consent for publication

No patients or human data used in this study. All authors read the manuscript and approved to be co-authors on the manuscript and have substantial contribution in the manuscript.

## Supplementary information

Supplementary Information.

## Data Availability

The datasets used and/or analysed during the current study are available from the corresponding author on a reasonable request. No human samples are used in this study. Mid brain containing *SNpc* region RNA-seq data raw data are available by authors as well as on GEO accession number (GSE 155739). The current manuscript has already been released on a pre-print server: https://www.biorxiv.org/content/10.1101/776005v1.
