# Supplementary material for: DJ-1 (Park7) affects the gut microbiome, metabolites and the development of innate lymphoid cells (ILCs)
Source: Sci Rep. 2020 Sep 30;10:16131. doi: 10.1038/s41598-020-72903-w (PMC7528091; doi:10.1038/s41598-020-72903-w)
Supplement: Supplementary file 1 — Supplementary Information. [file 41598_2020_72903_MOESM1_ESM.pdf]

DJ-1 (Park7) affects the gut microbiome, metabolites and the development of Innate Lymphoid cells (ILCs)

Yogesh Singh[1,4*], Christoph Trautwein[2], Achal Dhariwal[3], Madhuri S Salker[4], Md Alauddin[4], Laimdota Zizmare[2], Lisann Pelzl[5,6], Martina Feger[7], Jakob Admard[1], Nicolas Casadei[1], Michael Föller[7], Vivek Pachauri[8], David S Park[9], Tak W Mak[10], Julia-Stefanie Frick[11], Diethelm Wallwiener[4], Sara Y Brucker[4], Florian Lang[5#], Olaf Riess[1#]

*Address for correspondence

Yogesh Singh, PhD
Institute of Medical Genetics and Applied Genomics,
Calwerstraße 7,
University of Tübingen,
72076, Tübingen, Germany
Phone: +49 7071 29 72287
Fax: +49 7071 29 25098
Email: ysinghbt@gmail.com/yogesh.singh@med.uni-tuebingen.de

Short title: DJ-1 deficiency leads to gut dysbiosis

Suppl. Fig. legends

Suppl. Fig. 1: Species and functional pathway analysis.
(a) Clustering of all bacteria at species level. (b) Significant increase in *Rikenella sp.* and *Alistipes timonensis* in DJ-1$^{-/-}$ mice compared with DJ-1$^{+/+}$. Bar diagram (mean±SD) shows all the significantly different bacteria at species level in DJ-1$^{+/+}$ and DJ-1$^{-/-}$ mice. *represents the p value of <0.05 using Student's unpaired t-test.

Suppl. Fig. 2: KEGG functional gene pathway analysis from 16S rRNA.

Suppl. Fig. 3: Inflammatory cytokine production in the feces of DJ-1$^{-/-}$ and DJ-1$^{+/+}$ mice.

Suppl. Fig. 4: α-Syn and GFAP expression by IHC and Immunoblotting. (a) A representative IHCs colon pictures from DJ-1$^{+/+}$ (n=3) and DJ-1$^{-/-}$ (n=3) mice. Each mouse is shown and marked with #1, #2 and #3 and staining of α-Syn is shown with arrow heads in the pictures. (b) Bar diagram (mean±SEM) data shows similar expression in DJ-1$^{+/+}$ (n=5) and DJ-1$^{-/-}$ (n=5). (c) GFAP expression (green), nuclei (blue) in the colon as demonstrated by immunofluorescence staining.

Suppl. Fig. 5: NMR Metabolites Heatmap and metabolites clustering in DJ-1$^{+/+}$ and DJ-1$^{-/-}$ feces.

Suppl. Fig. 6: Example of ^{1}H-NMR spectra of DJ-1 and recorded reference standard spectrum for 3-HPPA

Suppl. Fig. 7: Original Immunoblots used in the figures. (A) GFAP and corresponding Gapdh. (B) α-Syn (mouse) and corresponding Gapdh.

Suppl. Table 1: Differentially expressed genes (DEGs) in DJ-1$^{-/-}$ mice mid brain containing *SNpc*.

Suppl. Table 2: Ingenuity canonical pathways in DJ-1$^{-/-}$ mice mid brain containing *SNpc*.

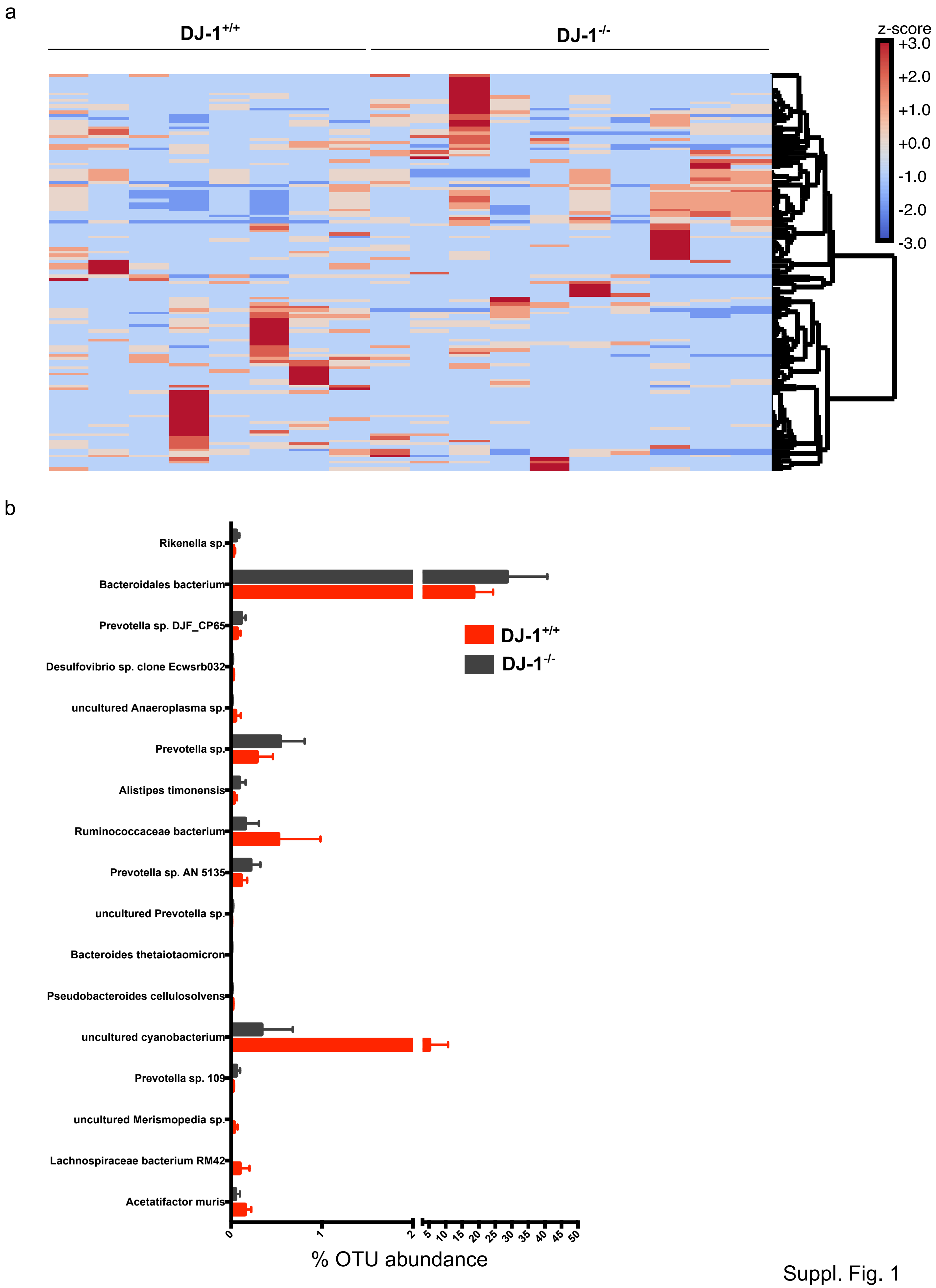
a
DJ-1+/+
DJ-1-/-
z-score
+3.0
+2.0
+1.0
+0.0
-1.0
-2.0
-3.0
b
Rikenella sp.
Bacteroidales bacterium
Prevotella sp. DJF_CP65
Desulfovibrio sp. clone Ecwsrb032
uncultured Anaeroplasma sp.
Prevotella sp.
Alistipes timonensis
Ruminococcaceae bacterium
Prevotella sp. AN 5135
uncultured Prevotella sp.
Bacteroides thetaiotaomicron
Pseudobacteroides cellulosolvens
uncultured cyanobacterium
Prevotella sp. 109
uncultured Merismopedia sp.
Lachnospiraceae bacterium RM42
Acetatifactor muris
DJ-1+/+
DJ-1-/-
0
1
2
5
10
15
20
25
30
35
40
45
50
% OTU abundance

Suppl. Fig. 1

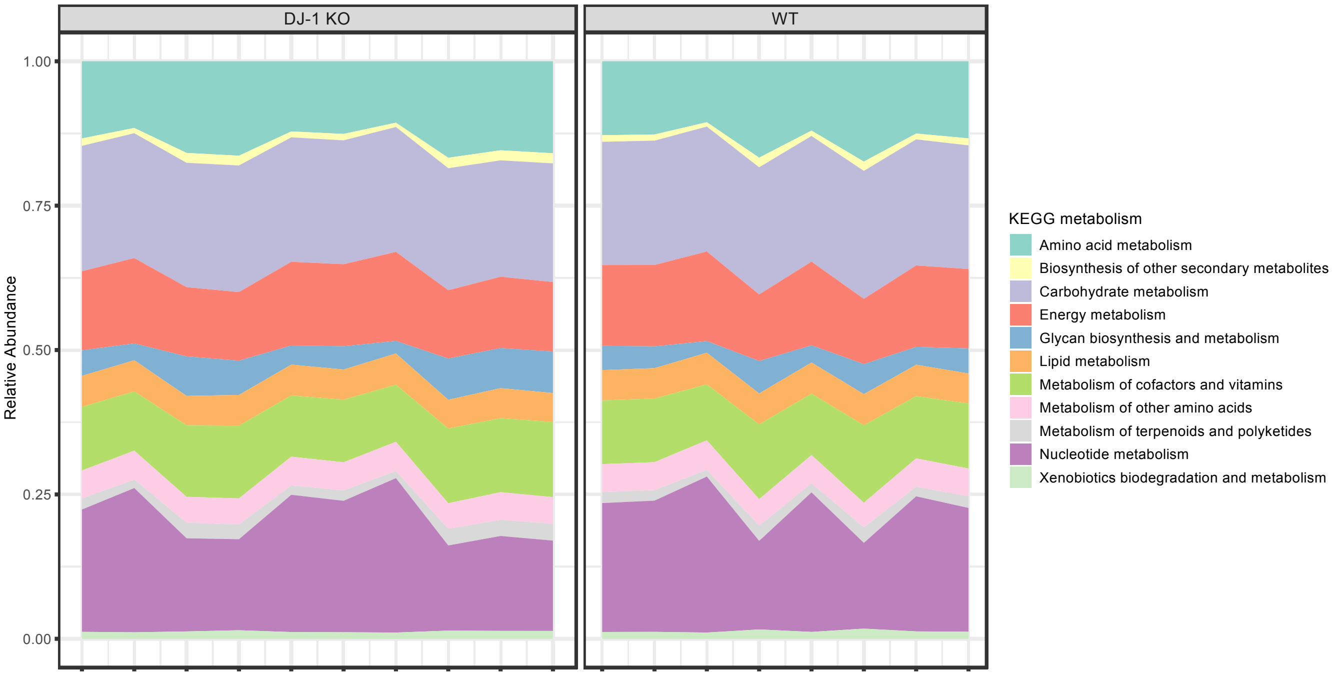
DJ-1 KO
WT
Relative Abundance
1.00
0.75
0.50
0.25
0.00
KEGG metabolism
Amino acid metabolism
Biosynthesis of other secondary metabolites
Carbohydrate metabolism
Energy metabolism
Glycan biosynthesis and metabolism
Lipid metabolism
Metabolism of cofactors and vitamins
Metabolism of other amino acids
Metabolism of terpenoids and polyketides
Nucleotide metabolism
Xenobiotics biodegradation and metabolism

Suppl. Fig. 2

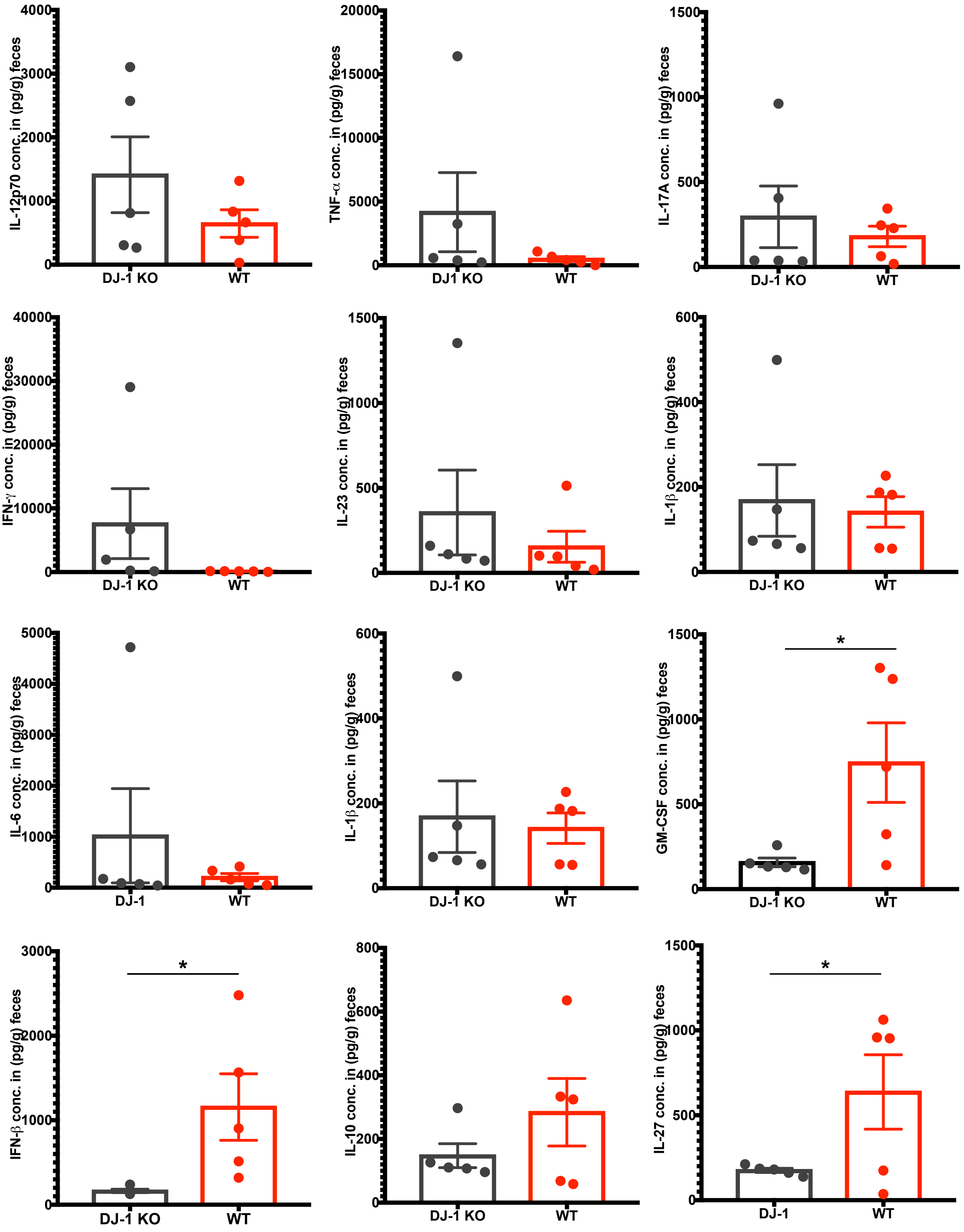

IL-12p70 conc. in (pg/g) feces
DJ-1 KO
WT
TNF-α conc. in (pg/g) feces
DJ1 KO
WT
IL-17A conc. in (pg/g) feces
DJ-1 KO
WT
IFN-γ conc. in (pg/g) feces
DJ-1 KO
WT
IL-23 conc. in (pg/g) feces
DJ-1 KO
WT
IL-1β conc. in (pg/g) feces
DJ-1 KO
WT
IL-6 conc. in (pg/g) feces
DJ-1
WT
IL-1β conc. in (pg/g) feces
DJ-1 KO
WT
GM-CSF conc. in (pg/g) feces
*
DJ-1 KO
WT
IFN-β conc. in (pg/g) feces
*
DJ-1 KO
WT
IL-10 conc. in (pg/g) feces
DJ-1 KO
WT
IL-27 conc. in (pg/g) feces
*
DJ-1
WT

Suppl. Fig. 3

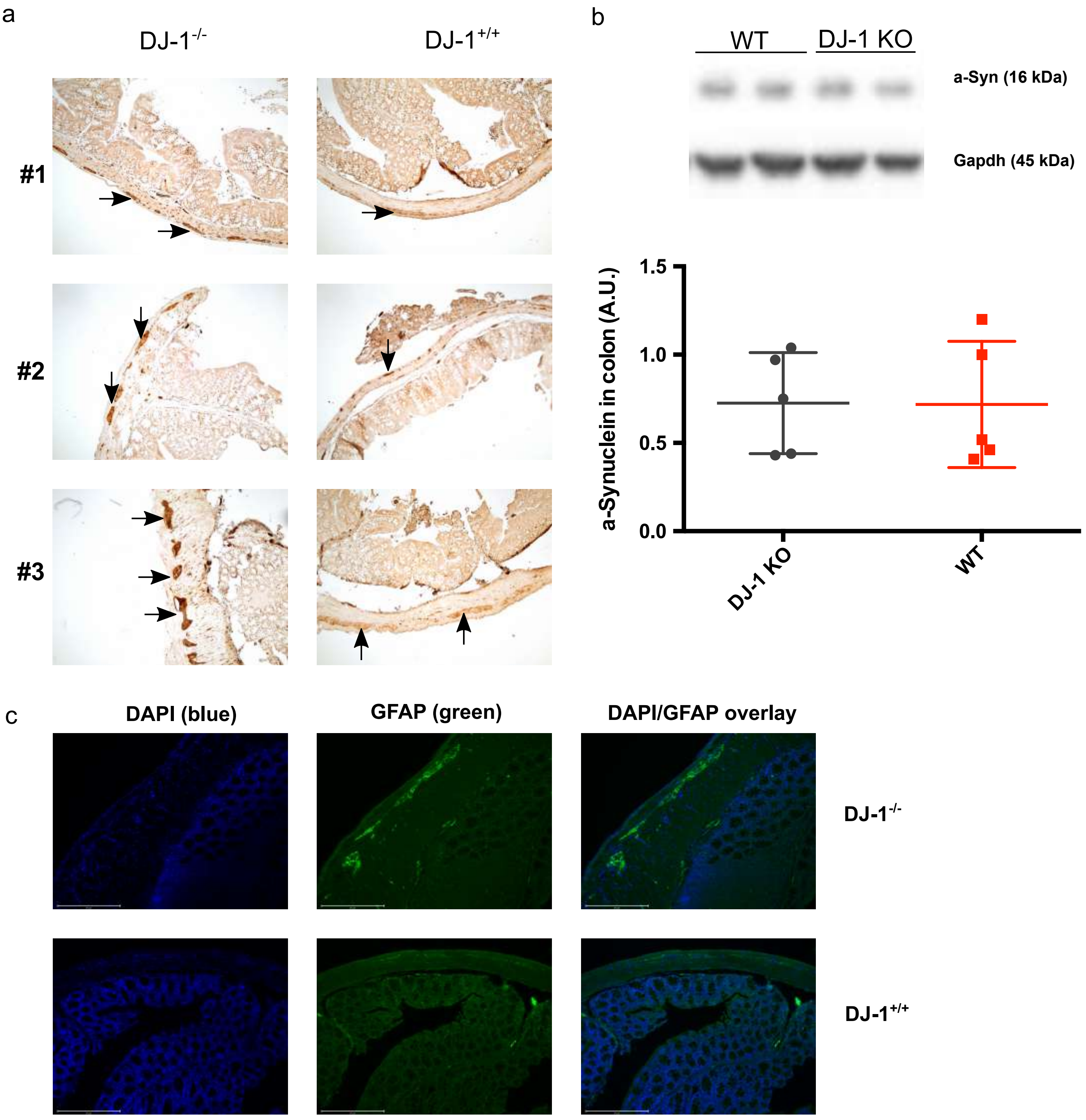

a
DJ-1-/-
DJ-1+/+
#1
#2
#3
b
WT
DJ-1 KO
a-Syn (16 kDa)
Gapdh (45 kDa)
a-Synuclein in colon (A.U.)
1.5
1.0
0.5
0.0
DJ-1 KO
WT
c
DAPI (blue)
GFAP (green)
DAPI/GFAP overlay
DJ-1-/-
DJ-1+/+

Suppl. Fig. 4

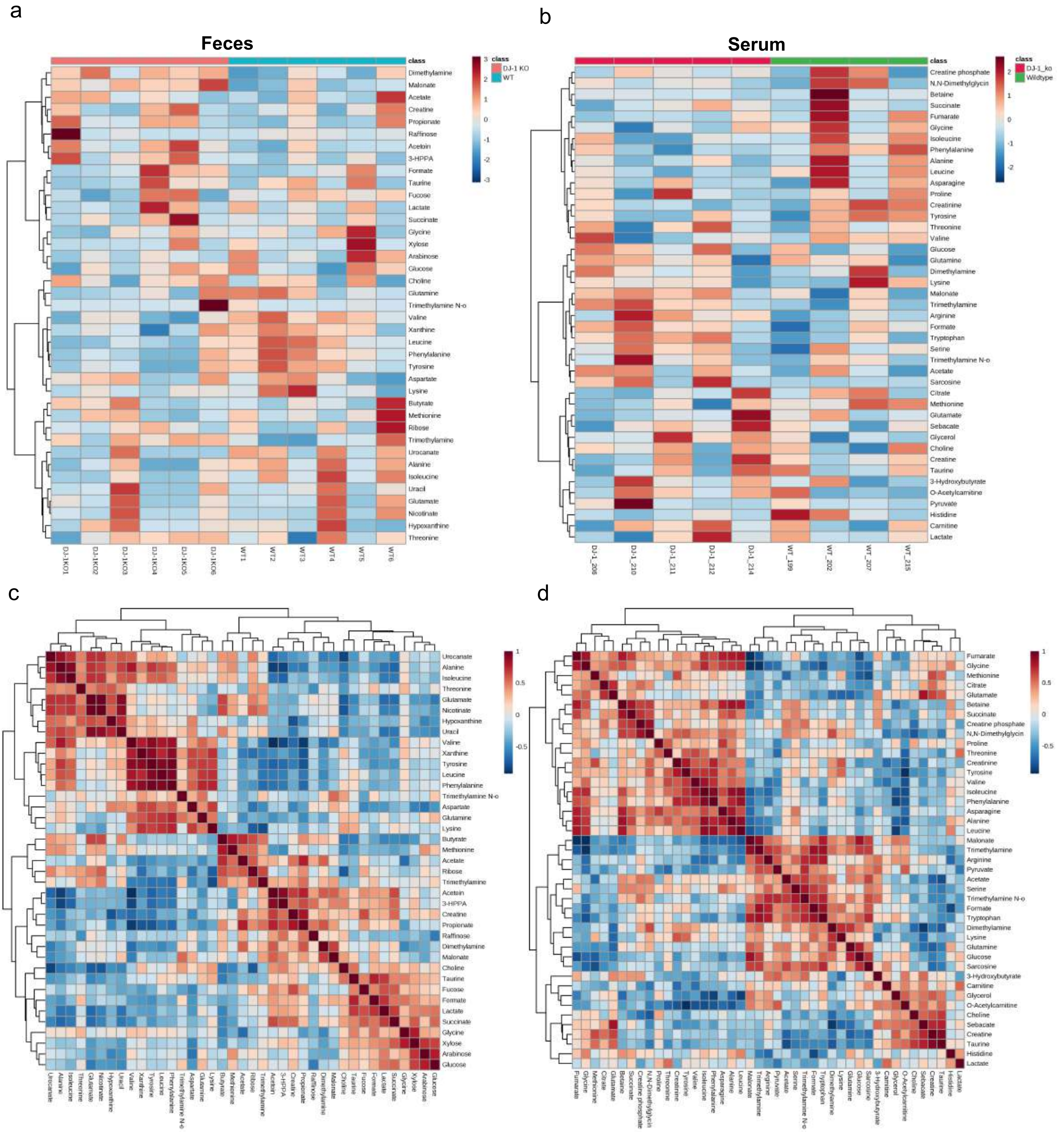

a
Feces
class
DJ-1 KO
WT
3
2
1
0
-1
-2
-3
Dimethylamine
Malonate
Acetate
Creatine
Propionate
Raffinose
Acetoin
3-HPPA
Formate
Taurine
Fucose
Lactate
Succinate
Glycine
Xylose
Arabinose
Glucose
Choline
Glutamine
Trimethylamine N-o
Valine
Xanthine
Leucine
Phenylalanine
Tyrosine
Aspartate
Lysine
Butyrate
Methionine
Ribose
Trimethylamine
Urocanate
Alanine
Isoleucine
Uracil
Glutamate
Nicotinate
Hypoxanthine
Threonine
DJ-1KO1
DJ-1KO2
DJ-1KO3
DJ-1KO4
DJ-1KO5
DJ-1KO6
WT1
WT2
WT3
WT4
WT5
WT6
b
Serum
class
DJ-1_ko
Wildtype
2
1
0
-1
-2
Creatine phosphate
N,N-Dimethylglycin
Betaine
Succinate
Fumarate
Glycine
Isoleucine
Phenylalanine
Alanine
Leucine
Asparagine
Proline
Creatinine
Tyrosine
Threonine
Valine
Glucose
Glutamine
Dimethylamine
Lysine
Malonate
Trimethylamine
Arginine
Formate
Tryptophan
Serine
Trimethylamine N-o
Acetate
Sarcosine
Citrate
Methionine
Glutamate
Sebacate
Glycerol
Choline
Creatine
Taurine
3-Hydroxybutyrate
O-Acetylcarnitine
Pyruvate
Histidine
Carnitine
Lactate
DJ-1_206
DJ-1_210
DJ-1_211
DJ-1_212
DJ-1_214
WT_199
WT_202
WT_207
WT_215
c
1
0.5
0
-0.5
Urocanate
Alanine
Isoleucine
Threonine
Glutamate
Nicotinate
Hypoxanthine
Uracil
Valine
Xanthine
Tyrosine
Leucine
Phenylalanine
Trimethylamine N-o
Aspartate
Glutamine
Lysine
Butyrate
Methionine
Acetate
Ribose
Trimethylamine
Acetoin
3-HPPA
Creatine
Propionate
Raffinose
Dimethylamine
Malonate
Choline
Taurine
Fucose
Formate
Lactate
Succinate
Glycine
Xylose
Arabinose
Glucose
d
1
0.5
0
-0.5
Fumarate
Glycine
Methionine
Citrate
Glutamate
Betaine
Succinate
Creatine phosphate
N,N-Dimethylglycin
Proline
Threonine
Creatinine
Tyrosine
Valine
Isoleucine
Phenylalanine
Asparagine
Alanine
Leucine
Malonate
Trimethylamine
Arginine
Pyruvate
Acetate
Serine
Trimethylamine N-o
Formate
Tryptophan
Dimethylamine
Lysine
Glutamine
Glucose
Sarcosine
3-Hydroxybutyrate
Carnitine
Glycerol
O-Acetylcarnitine
Choline
Sebacate
Creatine
Taurine
Histidine
Lactate

Suppl. Fig. 5

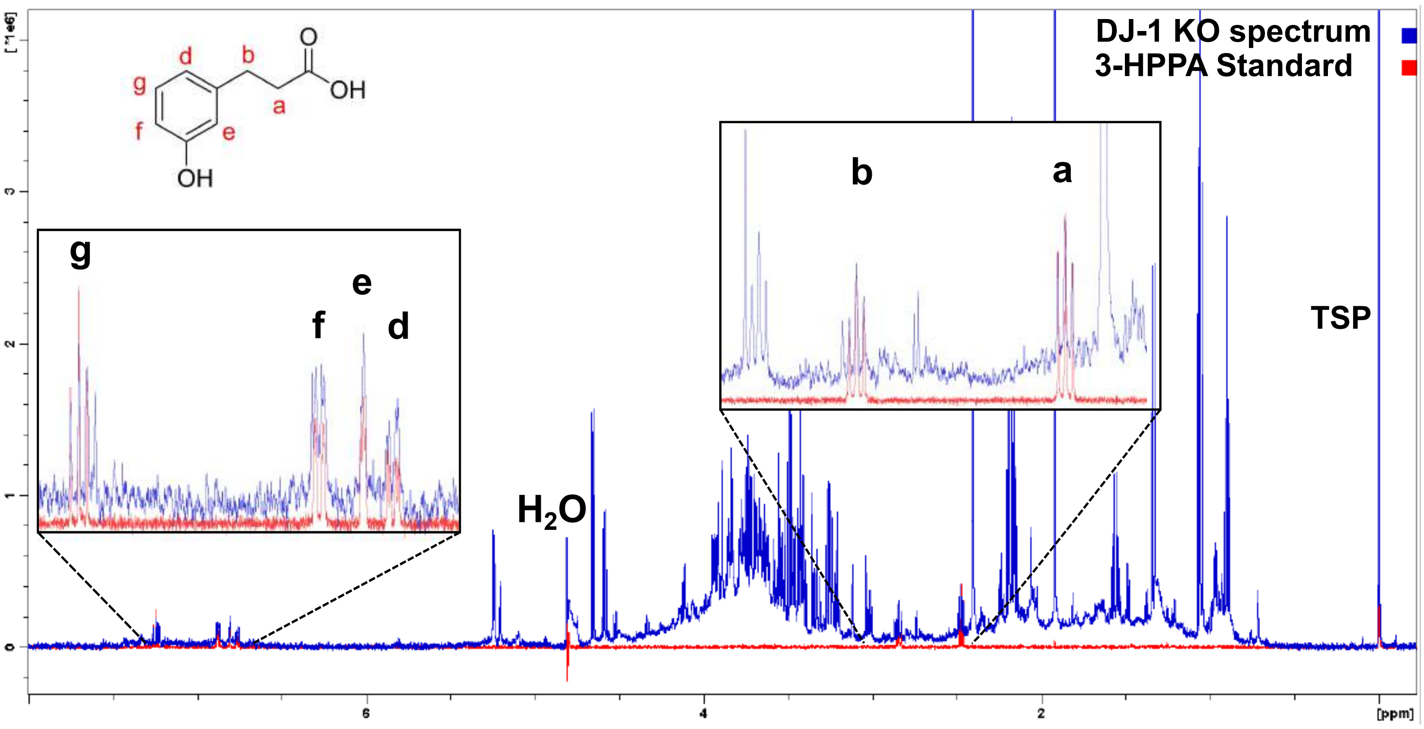

DJ-1 KO spectrum
3-HPPA Standard
OH
OH
d
b
a
g
f
e
g
f
e
d
b
a
H_2O
TSP
[*1e6]
[ppm]

Suppl. Fig. 6

a

Original WBs

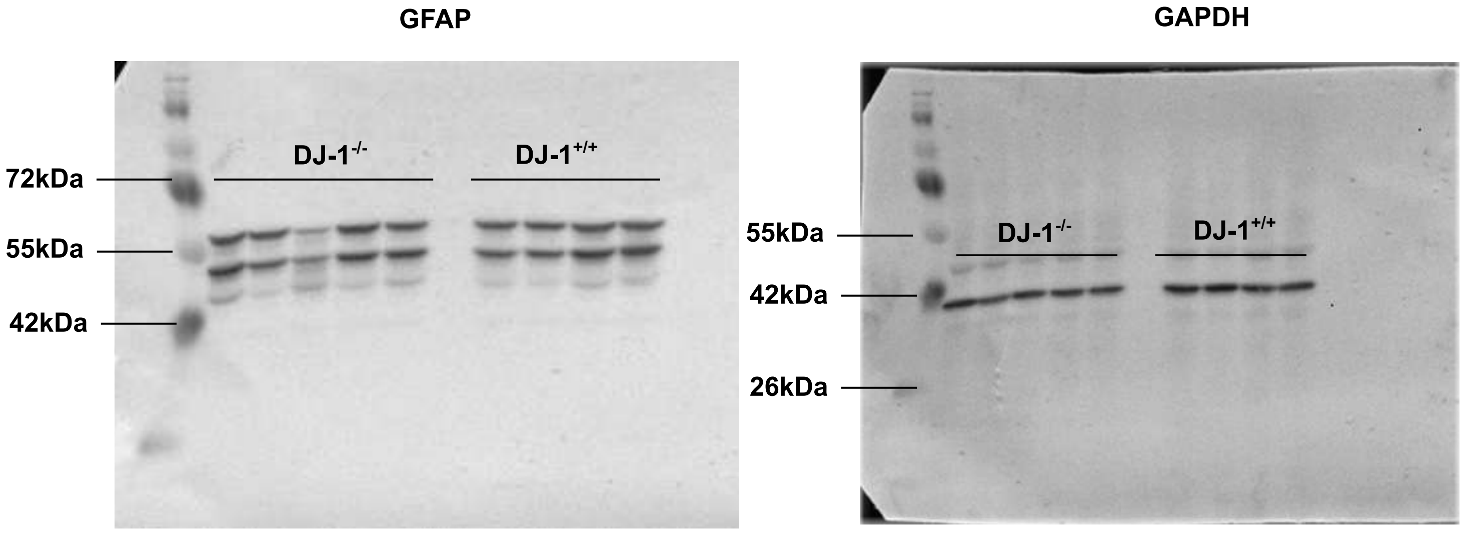

b

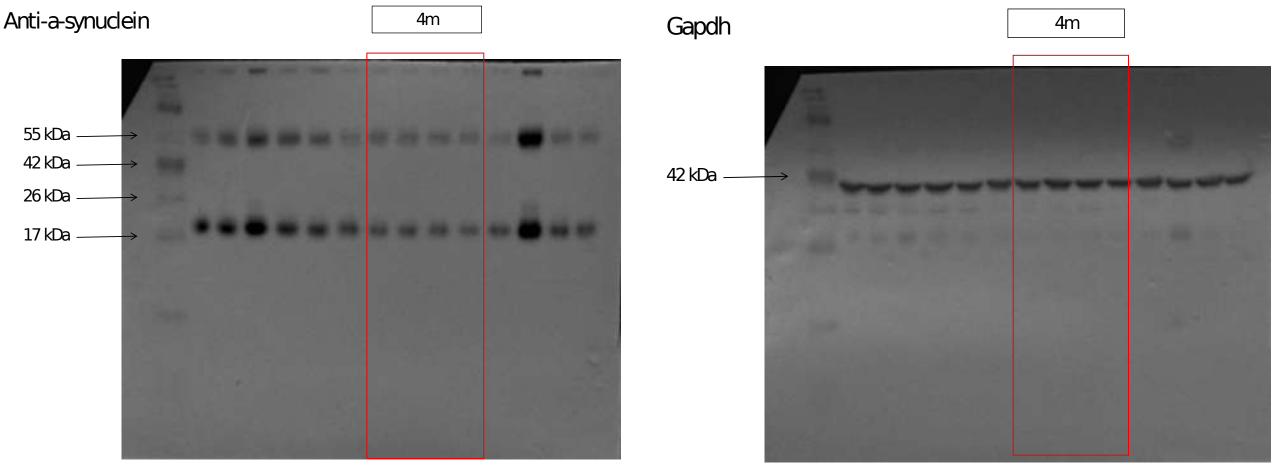

c

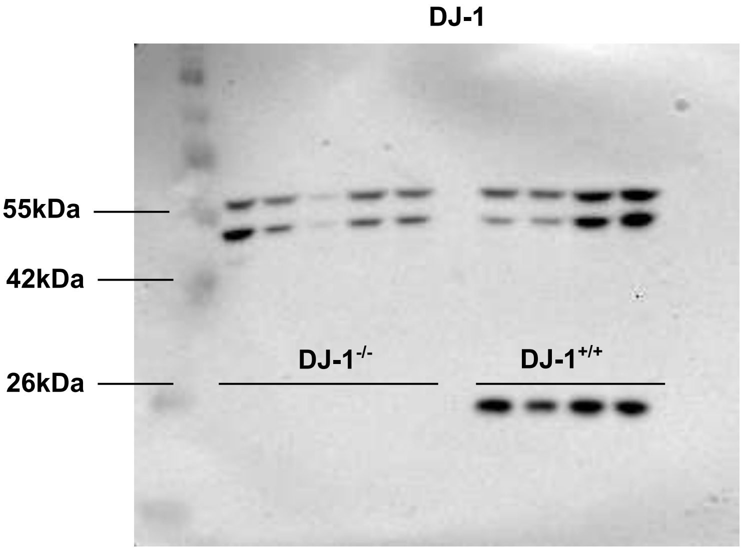

Suppl. Fig. 7

Suppl. Table 1

Samples

NameInternal	NameExternal	Group
20056a001_01	WT-1 (199)	wt
20056a002_01	WT-2 (202)	wt
20056a003_01	WT-3 (207)	wt
20056a004_01	WT-4 (215)	wt
20056a005_01	DJ-1 KO-1 (206)	ko
20056a006_01	DJ-1 KO-2 (210)	ko
20056a007_01	DJ-1 KO-3 ((211)	ko
20056a008_01	DJ-1 KO-4 (212)	ko
20056a009_01	DJ-1 KO-5 (214)	ko

Summary

Model: ~ Group
Filter: p-value < 0.05

Name	Description	up	down	total
ko	Knockout samples compare	275	254	529

Gene Overview

gene_id	symbol	ko	ko_logFC
ENSMUSG00000031075	Ano1	up	5.19
ENSMUSG00000005800	Mmp8	up	4.35
ENSMUSG00000033788	Dysf	up	4.21
ENSMUSG00000040681	Hmgn1	up	4.00
ENSMUSG00000085386	Gm13630	up	3.99
ENSMUSG00000064310	Zpld1	up	3.99
ENSMUSG00000026621	Marc1	up	3.85
ENSMUSG00000068220	Lgals1	up	3.66
ENSMUSG00000104445	Rhbg	up	3.13
ENSMUSG00000062319	Gm10115	up	3.12
ENSMUSG00000100484	Gm7133	up	2.97
ENSMUSG00000083087	Gm11249	up	2.88
ENSMUSG00000058290	Espl1	up	2.88
ENSMUSG00000106384	Gm43188	up	2.69
ENSMUSG00000104257	Gm20172	up	2.57
ENSMUSG00000109982	Gm45520	up	2.46
ENSMUSG00000105940	Gm42635	up	2.45
ENSMUSG00000062393	Dgkk	up	2.33
ENSMUSG00000099422	Gm4275	up	2.32
ENSMUSG00000108046	Gm43924	up	2.32
ENSMUSG00000048371	Pdp2	up	2.32
ENSMUSG00000093277	Gm26236	up	2.30
ENSMUSG00000059183	Mtfmt	up	2.30
ENSMUSG00000031503	Col4a2	up	2.29
ENSMUSG00000073209	Klf14	up	2.25
ENSMUSG00000005983	1700037C18Rik	up	2.22

ENSMUSG00000031078	Cttn	up	2.20
ENSMUSG00000067795	4930444P10Rik	up	2.18
ENSMUSG00000058486	Wdr91	up	2.18
ENSMUSG00000027224	Duoxa1	up	2.18
ENSMUSG00000110394	Gm18991	up	2.18
ENSMUSG00000060427	Zfp868	up	2.18
ENSMUSG00000100396	Gm29367	up	2.17
ENSMUSG00000040253	Gbp7	up	2.13
ENSMUSG00000049409	Prokr1	up	2.12
ENSMUSG00000105107	Gm43412	up	2.11
ENSMUSG00000113689	AC159239.2	up	2.04
ENSMUSG00000025876	Unc5a	up	2.03
ENSMUSG00000069301	Hist1h2ag	up	2.03
ENSMUSG00000060224	Pyroxd2	up	2.03
ENSMUSG00000036931	Nfkbid	up	2.03
ENSMUSG00000064609	Gm25635	up	2.02
ENSMUSG00000110465	Gm45832	up	1.96
ENSMUSG00000086000	Gm12493	up	1.95
ENSMUSG00000106707	Gm43748	up	1.95
ENSMUSG00000021609	Slc6a3	up	1.93
ENSMUSG00000085033	Gm11646	up	1.93
ENSMUSG00000114064	AC155260.1	up	1.93
ENSMUSG00000017204	Gsdma	up	1.92
ENSMUSG00000040521	Tsfm	up	1.92
ENSMUSG00000112304	AC158633.1	up	1.92
ENSMUSG00000040146	Rgl3	up	1.91
ENSMUSG00000055633	Zfp580	up	1.88
ENSMUSG00000014602	Kif1a	up	1.82
ENSMUSG00000036721	Zscan12	up	1.79
ENSMUSG00000026860	Sh3glb2	up	1.72
ENSMUSG00000091514	Gm17484	up	1.72
ENSMUSG00000074483	Bglap	up	1.68
ENSMUSG00000040447	Spns2	up	1.67
ENSMUSG00000110758	AC153729.1	up	1.66
ENSMUSG00000112597	AC153974.2	up	1.63
ENSMUSG00000086234	Usp46os2	up	1.63
ENSMUSG00000114907	AC173482.6	up	1.59
ENSMUSG00000108659	Gm34121	up	1.58
ENSMUSG00000114066	AC158354.2	up	1.57
ENSMUSG00000039628	Hs3st6	up	1.48
ENSMUSG00000031174	Rpgr	up	1.44
ENSMUSG00000036196	Slc26a8	up	1.40
ENSMUSG00000086206	Gm13783	up	1.39
ENSMUSG00000117982		up	1.36
ENSMUSG00000024295	Cyp4f41-ps	up	1.35
ENSMUSG00000073838	Tufm	up	1.34
ENSMUSG00000103059	Gm18186	up	1.32
ENSMUSG00000061374	Fiz1	up	1.29
ENSMUSG00000097885	5031434O11Rik	up	1.28
ENSMUSG00000006673	Qrich1	up	1.27
ENSMUSG00000104612	Gm42449	up	1.25
ENSMUSG00000059851	Kmt5c	up	1.24

ENSMUSG00000113022	CT025623.1	up	1.20
ENSMUSG00000109314	Gm44699	up	1.18
ENSMUSG00000108624	Gm45091	up	1.16
ENSMUSG00000037605	Adgrl3	up	1.16
ENSMUSG00000032563	Mrpl3	up	1.14
ENSMUSG00000032591	Mst1	up	1.13
ENSMUSG00000106856	Gm43761	up	1.10
ENSMUSG00000054764	Mtnr1a	up	1.10
ENSMUSG00000048388	Fam171b	up	1.09
ENSMUSG00000110455	Gm45904	up	1.08
ENSMUSG00000107232	1700017L05Rik	up	1.08
ENSMUSG00000020732	Rab37	up	1.07
ENSMUSG00000031661	Nkd1	up	1.07
ENSMUSG00000113842	AC122828.3	up	1.05
ENSMUSG00000109992	Gm45650	up	1.05
ENSMUSG00000019984	Med23	up	1.02
ENSMUSG00000027660	Skil	up	1.01
ENSMUSG00000026854	Usp20	up	0.98
ENSMUSG00000051000	Fam160a1	up	0.94
ENSMUSG00000060671	Atp8b2	up	0.93
ENSMUSG00000048234	Rnf149	up	0.93
ENSMUSG00000053897	Slc39a8	up	0.90
ENSMUSG00000030062	Rpn1	up	0.89
ENSMUSG00000003585	Sec14l2	up	0.89
ENSMUSG00000063434	Sorcs3	up	0.88
ENSMUSG00000080316	Spaca6	up	0.88
ENSMUSG00000049690	Nckap5	up	0.85
ENSMUSG00000035530	Eif1	up	0.85
ENSMUSG00000038600	Atp6v0a4	up	0.85
ENSMUSG00000103643	Gm37770	up	0.85
ENSMUSG00000114959	AC154314.2	up	0.83
ENSMUSG00000013367	Iglon5	up	0.83
ENSMUSG00000074217	2210011C24Rik	up	0.82
ENSMUSG00000027344	Fsip1	up	0.81
ENSMUSG00000094443	Sgo2b	up	0.80
ENSMUSG00000096957	E230013L22Rik	up	0.78
ENSMUSG00000020299	4930524B15Rik	up	0.77
ENSMUSG00000029674	Limk1	up	0.76
ENSMUSG00000040506	Ambra1	up	0.76
ENSMUSG00000050840	Cdh20	up	0.76
ENSMUSG00000051669	AU021092	up	0.76
ENSMUSG00000084093	Gm16418	up	0.73
ENSMUSG00000105282	Gm42981	up	0.72
ENSMUSG00000105352	C030018K13Rik	up	0.72
ENSMUSG00000030737	Slco2b1	up	0.71
ENSMUSG00000115624		up	0.71
ENSMUSG00000115599		up	0.70
ENSMUSG00000007080	Pole	up	0.68
ENSMUSG00000020526	Znhit3	up	0.68
ENSMUSG00000057182	Scn3a	up	0.68
ENSMUSG00000104974	Gm43686	up	0.67
ENSMUSG00000051224	Tceanc	up	0.67

ENSMUSG00000056019	Zfp709	up	0.65
ENSMUSG00000034203	Chchd4	up	0.65
ENSMUSG00000063870	Chd4	up	0.65
ENSMUSG00000035235	Trim13	up	0.65
ENSMUSG00000020700	Map3k3	up	0.65
ENSMUSG00000107698	Gm44430	up	0.65
ENSMUSG00000031388	Naa10	up	0.64
ENSMUSG00000104686	Gm42815	up	0.63
ENSMUSG00000110393	Gm36445	up	0.62
ENSMUSG00000004043	Stat5a	up	0.61
ENSMUSG00000006299	Aamp	up	0.61
ENSMUSG00000104410	Gm37066	up	0.61
ENSMUSG00000044824	Olfr545	up	0.60
ENSMUSG00000026411	Tmem9	up	0.60
ENSMUSG00000068243	Gm7079	up	0.59
ENSMUSG00000024909	Efemp2	up	0.59
ENSMUSG00000038342	Mlxip	up	0.59
ENSMUSG00000028433	Ubap2	up	0.58
ENSMUSG00000025147	Mob2	up	0.58
ENSMUSG00000046314	Stxbp6	up	0.58
ENSMUSG00000045917	Tmem268	up	0.57
ENSMUSG00000039747	Orai2	up	0.56
ENSMUSG00000112449	Srp54a	up	0.55
ENSMUSG00000117768		up	0.55
ENSMUSG00000042540	Acot5	up	0.55
ENSMUSG00000033569	Adgrb3	up	0.55
ENSMUSG00000110404	Rnf223	up	0.54
ENSMUSG00000029507	Pus1	up	0.53
ENSMUSG00000034893	Cog3	up	0.53
ENSMUSG00000032017	Grik4	up	0.52
ENSMUSG00000037638	Zbtb42	up	0.51
ENSMUSG00000022488	Nckap1l	up	0.51
ENSMUSG00000102139	Gm37109	up	0.51
ENSMUSG00000028443	Nudt2	up	0.50
ENSMUSG00000046111	Cep295	up	0.50
ENSMUSG00000008976	Gabpa	up	0.49
ENSMUSG00000112099	AC129329.1	up	0.49
ENSMUSG00000009628	Tex15	up	0.49
ENSMUSG00000021536	Adcy2	up	0.49
ENSMUSG00000012187	Mogat1	up	0.48
ENSMUSG00000024498	Tcerg1	up	0.48
ENSMUSG00000002076	Hsf2bp	up	0.48
ENSMUSG00000032456	Nmnat3	up	0.47
ENSMUSG00000107534	Gm43920	up	0.47
ENSMUSG00000060843	Ctnna3	up	0.47
ENSMUSG00000022748	Cmss1	up	0.47
ENSMUSG00000106802	Gm42885	up	0.46
ENSMUSG00000097023	Mir9-3hg	up	0.46
ENSMUSG00000025086	Trub1	up	0.46
ENSMUSG00000103996	4833421G17Rik	up	0.45
ENSMUSG00000107655	Gm44220	up	0.45
ENSMUSG00000100147	1700047M11Rik	up	0.44

ENSMUSG00000026496	Parp1	up	0.44
ENSMUSG00000034187	Nsf	up	0.43
ENSMUSG00000046727	Cystm1	up	0.43
ENSMUSG00000116919		up	0.43
ENSMUSG00000030741	Spns1	up	0.41
ENSMUSG00000059173	Pde1a	up	0.41
ENSMUSG00000095440	Fignl2	up	0.40
ENSMUSG00000026608	Kctd3	up	0.40
ENSMUSG00000020396	Nefh	up	0.40
ENSMUSG00000073236	2500004C02Rik	up	0.40
ENSMUSG00000078963	Hsbp1l1	up	0.39
ENSMUSG00000022664	Slc35a5	up	0.39
ENSMUSG00000024006	Stk38	up	0.39
ENSMUSG00000020952	Scfd1	up	0.39
ENSMUSG00000037544	Dlgap5	up	0.39
ENSMUSG00000117869		up	0.38
ENSMUSG00000114705	CT009536.2	up	0.38
ENSMUSG00000104379	Gm37509	up	0.38
ENSMUSG00000074261	Erich4	up	0.38
ENSMUSG00000078867	Gm14418	up	0.38
ENSMUSG00000028622	Mrpl37	up	0.38
ENSMUSG00000019961	Tmpo	up	0.37
ENSMUSG00000026339	Ccdc93	up	0.37
ENSMUSG00000090125	Pou3f1	up	0.37
ENSMUSG00000046658	Zfp316	up	0.37
ENSMUSG00000079108	Srp54b	up	0.36
ENSMUSG00000024084	Qpct	up	0.36
ENSMUSG00000104164	Gm38248	up	0.35
ENSMUSG00000020260	Pofut2	up	0.35
ENSMUSG00000021485	Mxd3	up	0.35
ENSMUSG00000030452	Nipa2	up	0.34
ENSMUSG00000031871	Cdh5	up	0.34
ENSMUSG00000025872	Thoc3	up	0.34
ENSMUSG00000020963	Tshr	up	0.34
ENSMUSG00000020407	Upp1	up	0.33
ENSMUSG00000044005	Gls2	up	0.32
ENSMUSG00000026283	Ing5	up	0.32
ENSMUSG00000091194	Gm19840	up	0.32
ENSMUSG00000078247	Airn	up	0.32
ENSMUSG00000055415	Atp10b	up	0.32
ENSMUSG00000085862	Gm13483	up	0.31
ENSMUSG00000029621	Arpc1a	up	0.31
ENSMUSG00000002055	Spag5	up	0.31
ENSMUSG00000020032	Nuak1	up	0.31
ENSMUSG00000097316	Gm10516	up	0.31
ENSMUSG00000116926		up	0.31
ENSMUSG00000084964	Gm15503	up	0.30
ENSMUSG00000050002	Idnk	up	0.30
ENSMUSG00000097156	Gm3764	up	0.30
ENSMUSG00000010122	Slc47a1	up	0.29
ENSMUSG00000083512	Gm12749	up	0.28
ENSMUSG00000033628	Pik3c3	up	0.28

ENSMUSG00000097823	Gm16701	up	0.28
ENSMUSG00000027091	Zc3h15	up	0.28
ENSMUSG00000021367	Edn1	up	0.28
ENSMUSG00000031958	Ldhd	up	0.27
ENSMUSG00000021007	Spata7	up	0.27
ENSMUSG00000039826	Trub2	up	0.26
ENSMUSG00000027722	Spata5	up	0.26
ENSMUSG00000049604	Hoxb13	up	0.26
ENSMUSG00000003534	Ddr1	up	0.25
ENSMUSG00000027673	Ndufb5	up	0.25
ENSMUSG00000117406		up	0.25
ENSMUSG00000098188	Sowahc	up	0.25
ENSMUSG00000022377	Asap1	up	0.24
ENSMUSG00000109080	Gm38944	up	0.24
ENSMUSG00000006724	Cyp27b1	up	0.24
ENSMUSG00000021469	Msx2	up	0.24
ENSMUSG00000105374	Gm42551	up	0.23
ENSMUSG00000037610	Kcnmb2	up	0.23
ENSMUSG00000020869	Lrrc59	up	0.23
ENSMUSG00000021998	Lcp1	up	0.23
ENSMUSG00000028221	Tmem55a	up	0.23
ENSMUSG00000103928	Gm37893	up	0.22
ENSMUSG00000098557	Kctd12	up	0.21
ENSMUSG00000021327	Zkscan3	up	0.21
ENSMUSG00000015002	Efr3a	up	0.21
ENSMUSG00000020123	Avpr1a	up	0.21
ENSMUSG00000002608	Ccdc97	up	0.21
ENSMUSG00000024030	Abcg1	up	0.20
ENSMUSG00000057409	Zfp53	up	0.20
ENSMUSG00000115012		up	0.20
ENSMUSG00000030465	Psd3	up	0.20
ENSMUSG00000044296	Zfp879	up	0.19
ENSMUSG00000102801	Gm37478	up	0.19
ENSMUSG00000060180	Myh13	up	0.19
ENSMUSG00000074652	Myh7b	up	0.18
ENSMUSG00000051879	Krt71	up	0.18
ENSMUSG00000096006	Gm21596	up	0.18
ENSMUSG00000090486	BC035947	up	0.17
ENSMUSG00000064653	Gm26129	up	0.17
ENSMUSG00000048895	Cdk5r1	up	0.17
ENSMUSG00000015702	Anxa9	up	0.16
ENSMUSG00000021965	Ska3	down	-0.15
ENSMUSG00000097525	Platr31	down	-0.16
ENSMUSG00000052981	Ube2ql1	down	-0.16
ENSMUSG00000038250	Usp38	down	-0.17
ENSMUSG00000062075	Lmnb2	down	-0.17
ENSMUSG00000034083	Ccdc174	down	-0.18
ENSMUSG00000031027	Stk33	down	-0.18
ENSMUSG00000086944	Gm15859	down	-0.18
ENSMUSG00000091931	Gon7	down	-0.18
ENSMUSG00000004730	Adgre1	down	-0.18
ENSMUSG00000036561	Ppp6r2	down	-0.18

ENSMUSG00000021697	Depdc1b	down	-0.18
ENSMUSG00000042155	Klhl23	down	-0.18
ENSMUSG00000067872	Ccdc87	down	-0.19
ENSMUSG00000039686	Zer1	down	-0.19
ENSMUSG00000019851	Perp	down	-0.19
ENSMUSG00000026036	Nif3l1	down	-0.19
ENSMUSG00000028436	Dcaf12	down	-0.19
ENSMUSG00000052387	Trpm3	down	-0.19
ENSMUSG00000027380	Acoxl	down	-0.19
ENSMUSG00000083844	Ube2d-ps	down	-0.19
ENSMUSG00000042211	Fbxo38	down	-0.19
ENSMUSG00000020235	Fzr1	down	-0.19
ENSMUSG00000055301	Adh7	down	-0.19
ENSMUSG00000115338		down	-0.20
ENSMUSG00000062433	Krtap6-2	down	-0.20
ENSMUSG00000058835	Abi1	down	-0.20
ENSMUSG00000097164	Cep83os	down	-0.20
ENSMUSG00000090291	Lrrc10b	down	-0.20
ENSMUSG00000024845	Tmem134	down	-0.20
ENSMUSG00000035840	Lysmd3	down	-0.20
ENSMUSG00000068205	Macrod2	down	-0.20
ENSMUSG00000115264		down	-0.20
ENSMUSG00000026697	Myoc	down	-0.20
ENSMUSG00000028226	Mmp16	down	-0.21
ENSMUSG00000001436	Slc19a1	down	-0.21
ENSMUSG00000021903	Galnt15	down	-0.21
ENSMUSG00000069539	Scyl2	down	-0.21
ENSMUSG00000113903	CT485612.11	down	-0.21
ENSMUSG00000019774	Mtrf1l	down	-0.21
ENSMUSG00000028369	Svep1	down	-0.21
ENSMUSG00000019718	L3hypdh	down	-0.21
ENSMUSG00000003549	Ercc1	down	-0.21
ENSMUSG00000032942	Ucp3	down	-0.21
ENSMUSG00000034317	Trim59	down	-0.21
ENSMUSG00000038550	Ciart	down	-0.21
ENSMUSG00000043019	Edem3	down	-0.21
ENSMUSG00000020850	Prpf8	down	-0.21
ENSMUSG00000035722	Abca7	down	-0.22
ENSMUSG00000038150	Ormdl3	down	-0.22
ENSMUSG00000018322	Tomm34	down	-0.22
ENSMUSG00000069844	Sco1	down	-0.22
ENSMUSG00000054312	Mrps21	down	-0.22
ENSMUSG00000020519	Sap30l	down	-0.22
ENSMUSG00000020062	Slc5a8	down	-0.22
ENSMUSG00000020124	Usp15	down	-0.22
ENSMUSG00000069814	Ccdc92b	down	-0.23
ENSMUSG00000027669	Gnb4	down	-0.23
ENSMUSG00000069893	9930111J21Rik1	down	-0.23
ENSMUSG00000039097	Rln1	down	-0.24
ENSMUSG00000085845	Gm13944	down	-0.24
ENSMUSG00000041731	Pgm5	down	-0.24
ENSMUSG00000118274		down	-0.24

ENSMUSG00000099874	Gm29629	down	-0.24
ENSMUSG00000071054	Safb	down	-0.24
ENSMUSG00000025925	Terf1	down	-0.24
ENSMUSG00000025609	Mkln1	down	-0.24
ENSMUSG00000050737	Ptges	down	-0.24
ENSMUSG00000028199	Cryz	down	-0.24
ENSMUSG00000038128	Camk4	down	-0.25
ENSMUSG00000020799	Tekt1	down	-0.25
ENSMUSG00000062198	2700097O09Rik	down	-0.25
ENSMUSG00000020844	Nxn	down	-0.25
ENSMUSG00000040841	Six5	down	-0.25
ENSMUSG00000045763	Basp1	down	-0.25
ENSMUSG00000038871	Bpgm	down	-0.25
ENSMUSG00000054728	Phactr1	down	-0.25
ENSMUSG00000071660	Ttc9c	down	-0.25
ENSMUSG00000020736	Nt5c	down	-0.26
ENSMUSG00000035890	Rnf126	down	-0.26
ENSMUSG00000037935	Smarce1	down	-0.26
ENSMUSG00000000296	Tpd52l1	down	-0.26
ENSMUSG00000071713	Csf2rb	down	-0.26
ENSMUSG00000104362	Gm37928	down	-0.26
ENSMUSG00000022594	Lynx1	down	-0.26
ENSMUSG00000028016	Ints12	down	-0.27
ENSMUSG00000060923	Acyp2	down	-0.27
ENSMUSG00000015599	Ttbk1	down	-0.27
ENSMUSG00000069094	Pde7a	down	-0.27
ENSMUSG00000079259	Trim71	down	-0.27
ENSMUSG00000065922	n-R5-8s1	down	-0.27
ENSMUSG00000047787	Flrt1	down	-0.27
ENSMUSG00000025816	Sec61a2	down	-0.27
ENSMUSG00000025102	3110040N11Rik	down	-0.27
ENSMUSG00000085067	Gm15631	down	-0.27
ENSMUSG00000067158	Col4a4	down	-0.28
ENSMUSG00000077350	n-R5s31	down	-0.28
ENSMUSG00000071356	Reg3b	down	-0.29
ENSMUSG00000116348		down	-0.29
ENSMUSG00000085176	Gm15397	down	-0.30
ENSMUSG00000018761	Mpdu1	down	-0.31
ENSMUSG00000028524	Sgip1	down	-0.31
ENSMUSG00000105636	Gm43625	down	-0.32
ENSMUSG00000005553	Atp4a	down	-0.33
ENSMUSG00000097804	Gm16685	down	-0.33
ENSMUSG00000103713	Gm2136	down	-0.34
ENSMUSG00000021565	Slc6a19	down	-0.34
ENSMUSG00000022565	Plec	down	-0.34
ENSMUSG00000024906	Mus81	down	-0.34
ENSMUSG00000040966	Slc22a2	down	-0.34
ENSMUSG00000007872	Id3	down	-0.34
ENSMUSG00000010721	Lmbr1	down	-0.34
ENSMUSG00000004667	Polr2e	down	-0.35
ENSMUSG00000030982	9030624J02Rik	down	-0.37
ENSMUSG00000040987	Mill2	down	-0.37

ENSMUSG00000078808	Vmn1r58	down	-0.38
ENSMUSG00000002833	Hdgfl2	down	-0.39
ENSMUSG00000024427	Spry4	down	-0.40
ENSMUSG00000116597		down	-0.41
ENSMUSG00000108798	Gm9521	down	-0.42
ENSMUSG00000030806	Stx1b	down	-0.44
ENSMUSG00000030726	Pold3	down	-0.45
ENSMUSG00000003382	Etv3	down	-0.45
ENSMUSG00000104969	Gm43445	down	-0.49
ENSMUSG00000022658	Tagln3	down	-0.50
ENSMUSG00000118207		down	-0.51
ENSMUSG00000028776	Tinagl1	down	-0.51
ENSMUSG00000105906	Iglc1	down	-0.52
ENSMUSG00000042102	Dmgdh	down	-0.53
ENSMUSG00000004344	Gpx5	down	-0.54
ENSMUSG00000054423	Cadps	down	-0.56
ENSMUSG00000089941	Gm16168	down	-0.56
ENSMUSG00000041891	Lman1	down	-0.57
ENSMUSG00000079465	Col4a3	down	-0.58
ENSMUSG00000030666	Calcb	down	-0.60
ENSMUSG00000028609	Magoh	down	-0.61
ENSMUSG00000042788	Fam166b	down	-0.61
ENSMUSG00000042167	Papd4	down	-0.64
ENSMUSG00000073489	Ifi204	down	-0.64
ENSMUSG00000102309	Gm38189	down	-0.64
ENSMUSG00000038840	Birc7	down	-0.66
ENSMUSG00000099034	2810039B14Rik	down	-0.68
ENSMUSG00000073295	Nudt11	down	-0.69
ENSMUSG00000027487	Cdk5rap1	down	-0.69
ENSMUSG00000105021	Gm8234	down	-0.70
ENSMUSG00000020152	Actr2	down	-0.73
ENSMUSG00000021099	Six6	down	-0.79
ENSMUSG00000057614	Gnai1	down	-0.80
ENSMUSG00000002550	Uck1	down	-0.87
ENSMUSG00000117350		down	-0.88
ENSMUSG00000112481	AC155710.1	down	-0.88
ENSMUSG00000105294	Gm43304	down	-0.89
ENSMUSG00000052403	Fcnaos	down	-0.91
ENSMUSG00000097456	Gm16958	down	-0.92
ENSMUSG00000028991	Mtor	down	-0.93
ENSMUSG00000003435	Supt5	down	-0.94
ENSMUSG00000097442	Gm26632	down	-0.94
ENSMUSG00000030747	Dgat2	down	-0.94
ENSMUSG00000027997	Casp6	down	-0.96
ENSMUSG00000000686	Abhd15	down	-0.96
ENSMUSG00000037086	Prr32	down	-0.99
ENSMUSG00000033323	Ctdp1	down	-1.00
ENSMUSG00000083142	Gm12288	down	-1.00
ENSMUSG00000006763	Saal1	down	-1.05
ENSMUSG00000015013	Trappc2l	down	-1.05
ENSMUSG00000064585	Gm25129	down	-1.06
ENSMUSG00000073007	Fam46d	down	-1.10

ENSMUSG00000048411	Gm597	down	-1.13
ENSMUSG00000006154	Eps8l1	down	-1.13
ENSMUSG00000031176	Dynlt3	down	-1.18
ENSMUSG00000079410	Gm2897	down	-1.18
ENSMUSG00000044442	N6amt1	down	-1.18
ENSMUSG00000025350	Rdh5	down	-1.18
ENSMUSG00000003420	Fcgrt	down	-1.21
ENSMUSG00000110065	Gm45588	down	-1.23
ENSMUSG00000034112	Atp2c2	down	-1.24
ENSMUSG00000089679	Gm16299	down	-1.25
ENSMUSG00000112941	AC164441.1	down	-1.26
ENSMUSG00000028648	Ndufs5	down	-1.29
ENSMUSG00000063129	Aldoart2	down	-1.30
ENSMUSG00000061288	Taok3	down	-1.34
ENSMUSG00000117910		down	-1.36
ENSMUSG00000029068	Ccnl2	down	-1.36
ENSMUSG00000085316	D330050G23Rik	down	-1.38
ENSMUSG00000111834	AL928687.1	down	-1.41
ENSMUSG00000071604	Fam189a2	down	-1.42
ENSMUSG00000000628	Hk2	down	-1.46
ENSMUSG00000029238	Clock	down	-1.47
ENSMUSG00000035168	Tanc1	down	-1.47
ENSMUSG00000030785	Cox6a2	down	-1.49
ENSMUSG00000029575	Mmab	down	-1.49
ENSMUSG00000078580	E430018J23Rik	down	-1.49
ENSMUSG00000029670	Ing3	down	-1.49
ENSMUSG00000064063	BC048507	down	-1.50
ENSMUSG00000023031	Cela1	down	-1.56
ENSMUSG00000109127	Gm31135	down	-1.56
ENSMUSG00000033904	Ccp110	down	-1.62
ENSMUSG00000048355	Arxes1	down	-1.63
ENSMUSG00000021012	Zc3h14	down	-1.63
ENSMUSG00000109727	Gm45464	down	-1.64
ENSMUSG00000025534	Gusb	down	-1.65
ENSMUSG00000091735	Gpr62	down	-1.71
ENSMUSG00000026098	Pms1	down	-1.72
ENSMUSG00000045010	Gm4779	down	-1.80
ENSMUSG00000033227	Wnt6	down	-1.83
ENSMUSG00000069188	Gm13192	down	-1.85
ENSMUSG00000024665	Fads2	down	-1.85
ENSMUSG00000009108	Gnat2	down	-1.85
ENSMUSG00000080773	Gm12955	down	-1.85
ENSMUSG00000029020	Mfn2	down	-1.97
ENSMUSG00000026473	Glul	down	-2.00
ENSMUSG00000029056	Pank4	down	-2.01
ENSMUSG00000086953	Aknaos	down	-2.03
ENSMUSG00000040283	Btnl9	down	-2.03
ENSMUSG00000025014	Dntt	down	-2.03
ENSMUSG00000105528	Gm43519	down	-2.05
ENSMUSG00000103011	Gm8860	down	-2.07
ENSMUSG00000052698	Tln2	down	-2.07
ENSMUSG00000109628	BC024386	down	-2.08

ENSMUSG00000098854	Gm5118	down	-2.08
ENSMUSG00000060882	Kcnd2	down	-2.11
ENSMUSG00000021972	Hmbox1	down	-2.19
ENSMUSG00000041707	1810011H11Rik	down	-2.19
ENSMUSG00000086679	Gm15551	down	-2.20
ENSMUSG00000031154	Otud5	down	-2.20
ENSMUSG00000093073	Mir124a-2	down	-2.21
ENSMUSG00000029992	Gfpt1	down	-2.23
ENSMUSG00000099869	1700030F04Rik	down	-2.26
ENSMUSG00000100862	Gm10925	down	-2.26
ENSMUSG00000045414	1190002N15Rik	down	-2.26
ENSMUSG00000016409	Nkap	down	-2.26
ENSMUSG00000081059	Gm11945	down	-2.26
ENSMUSG00000006262	Mob1b	down	-2.27
ENSMUSG00000074384	AI429214	down	-2.33
ENSMUSG00000014470	Rnf166	down	-2.38
ENSMUSG00000024603	Dctn4	down	-2.46
ENSMUSG00000073481	Marc2	down	-2.46
ENSMUSG00000109055	Gm2676	down	-2.50
ENSMUSG00000003657	Calb2	down	-2.50
ENSMUSG00000033152	Podxl2	down	-2.50
ENSMUSG00000052295	8030423F21Rik	down	-2.50
ENSMUSG00000007646	Rad51c	down	-2.52
ENSMUSG00000118087		down	-2.53
ENSMUSG00000112023	Lilr4b	down	-2.71
ENSMUSG00000005846	Rsl1d1	down	-2.72
ENSMUSG00000042613	Pbxip1	down	-2.76
ENSMUSG00000086238	Gm14258	down	-2.89
ENSMUSG00000026356	Dars	down	-3.27
ENSMUSG00000085222	Gm13974	down	-3.29
ENSMUSG00000090381	Gm6158	down	-3.29
ENSMUSG00000114218	AC159195.1	down	-3.55
ENSMUSG00000035354	Uvrag	down	-3.58
ENSMUSG00000085541	Gm16010	down	-3.82
ENSMUSG00000048000	Gigyf2	down	-4.46

Suppl. Table 2

Ingenuity Canonical Pathways	-log(p-value	Ratio	z-score	Molecules
**Upregulated pathways**				
Signaling by Rho Family GTPases	1,82E00	2,87E-02	1,342	ACTR2,ARPC1A,CDH20,CDH5,GNAI1,GNAT2,LIMK1
Cardiac Hypertrophy Signaling	9,59E-01	2,08E-02	1,000	ADCY2,GNAI1,GNAT2,MAP3K3,MTOR
Sirtuin Signaling Pathway	4,37E-01	1,37E-02	1,000	CLOCK,GABPA,MTOR,PARP1
Insulin Secretion Signaling Pathway	1,36E00	2,47E-02	0,816	ADCY2,Arxes1/Arxes2,MTOR,NSF,SRP54,STAT5A
Estrogen Receptor Signaling	1,23E00	2,13E-02	0,378	ADCY2,GNAI1,GNAT2,LIMK1,MED23,MMP8,MTOR
**Downregulated pathways**				
Ephrin Receptor Signaling	1,38E00	2,78E-02	-0,447	ACTR2,ARPC1A,GNAI1,GNAT2,LIMK1
fMLP Signaling in Neutrophils	1,47E00	3,45E-02	-1,000	ACTR2,ARPC1A,GNAI1,NFKBID
Endocannabinoid Cancer Inhibition Pathway	1,19E00	2,8E-02	-1,000	ADCY2,CASP6,GNAI1,MTOR
RhoGDI Signaling	2,52E00	3,89E-02	-1,342	ACTR2,ARPC1A,CDH20,CDH5,GNAI1,GNAT2,LIMK1
Endothelin-1 Signaling	8,63E-01	2,13E-02	-2,000	ADCY2,CASP6,GNAI1,GNAT2
Hepatic Fibrosis Signaling Pathway	2,65E-01	1,09E-02	-2,000	GNAI1,MTOR,NFKBID,WNT6
**No change in pathways but a trend**				
Synaptogenesis Signaling Pathway	2,74E00	3,21E-02	0,000	ACTR2,ADCY2,ARPC1A,CDH20,CDH5,LIMK1,MTOR,NSF,STX1B,STXBP6
Death Receptor Signaling	1,81E00	4,4E-02	0,000	CASP6,LIMK1,NFKBID,PARP1
NER Pathway	1,63E00	3,88E-02	0,000	HMGN1,POLD3,POLE,POLR2E
Reelin Signaling in Neurons	1,32E00	3,1E-02	0,000	ACTR2,ARPC1A,LIMK1,MTOR
Leukocyte Extravasation Signaling	1,24E00	2,54E-02	0,000	CDH5,CTNNA3,CTTN,GNAI1,MMP8
Actin Cytoskeleton Signaling	1,09E00	2,29E-02	0,000	ACTR2,ARPC1A,LIMK1,NCKAP1L,TLN2
Natural Killer Cell Signaling	8,12E-01	2,03E-02	0,000	LIMK1,MAP3K3,MICB,MTOR
HIF1α Signaling	7,7E-01	1,95E-02	0,000	HK2,MMP8,MTOR,NAA10
Integrin Signaling	7,28E-01	1,88E-02	0,000	ACTR2,ARPC1A,CTTN,TLN2
Cardiac Hypertrophy Signaling (Enhanced)	3,93E-01	1,23E-02	0,000	ADCY2,GNAI1,MAP3K3,MTOR,PDE1A,WNT6
Neuroinflammation Signaling Pathway	0.00E+00	1.00E-02	0,000	BIRC7,CALB2,GLUL
Glutamine Biosynthesis I	1,98E00	1.00E+00	0,000	GLUL
IL-1 Signaling	1,81E00	4,4E-02	0,000	ADCY2,GNAI1,GNAT2,NFKBID
Glutamine Degradation I	1,68E00	5.00E-01	0,000	GLS2
